# Identification of Crucial Parameters in a Mathematical Multiscale Model of Glioblastoma Growth

**DOI:** 10.1155/2014/437094

**Published:** 2014-05-08

**Authors:** Tina A. Schuetz, Andreas Mang, Stefan Becker, Alina Toma, Thorsten M. Buzug

**Affiliations:** ^1^Institute of Medical Engineering, University of Luebeck, Ratzeburger Allee 160, 23562 Luebeck, Germany; ^2^Graduate School for Computing in Medicine and Life Sciences, University of Luebeck, Ratzeburger Allee 160, 23562 Luebeck, Germany; ^3^Institute for Computational Engineering and Sciences, University of Texas at Austin, 201 East 24th Street, Stop C0200, Austin, TX 78712-1229, USA; ^4^Centre of Excellence for Technology and Engineering in Medicine (TANDEM), Ratzeburger Allee 160, 23562 Luebeck, Germany

## Abstract

Glioblastomas are highly malignant brain tumours. Mathematical models and their analysis provide a tool to support the understanding of the development of these tumours as well as the design of more effective treatment strategies. We have previously developed a multiscale model of glioblastoma progression that covers processes on the cellular and molecular scale. Here, we present a novel nutrient-dependent multiscale sensitivity analysis of this model that helps to identify those reaction parameters of the molecular interaction network that influence the tumour progression on the cellular scale the most. In particular, those parameters are identified that essentially determine tumour expansion and could be therefore used as potential therapy targets. As indicators for the success of a potential therapy target, a deceleration of the tumour expansion and a reduction of the tumour volume are employed. From the results, it can be concluded that no single parameter variation results in a less aggressive tumour. However, it can be shown that a few combined perturbations of two systematically selected parameters cause a slow-down of the tumour expansion velocity accompanied with a decrease of the tumour volume. Those parameters are primarily linked to the reactions that involve the microRNA-451 and the thereof regulated protein MO25.

## 1. Introduction

The glioblastoma (GB) still can be considered to be the most aggressive primary brain tumour [1] in humans. The current standard therapy is composed of a resection (if possible) and a combined radio- and chemotherapy [2]. However, the current median survival is only about 12 months [3]. Only 4.7% of all patients are still alive five years after diagnosis [4]. Therefore, current research focuses on gaining a better understanding of the disease and developing new treatment options. Among others, several relevant mutations and signalling pathways for GB emergence and progression have been identified and are one of the primary fields of research [5]. Consequently, the efficacy of molecular targeted drugs for GB treatment has been the focus of recent investigations. Yet, in practice, none of the currently available molecular targeted drugs resulted in a significant improvement of the survival expectancy [2].

In recent years, the significance of microRNAs and the thereof dependent signalling pathways for cancer has been discovered [6–8]. MicroRNAs are noncoding RNAs that influence the expression of proteins on the posttranscriptional level [6]. In [9], the role of microRNA-451 (miR-451) in glioblastoma is discussed. Godlewski et al. discovered that, on the one hand, in glioblastoma, the extracellular glucose concentration influences the level of miR-451. On the other hand, miR-451 regulates the signalling of the (well-known) LKB1-AMPK-mTOR pathway (LKB1: liver kinase B1, AMPK: AMP activated kinase, and mTOR: mammalian target of rapamycin) [10, 11]. This intracellular pathway is considerably involved in a cell's decision for either a migrating or proliferating phenotype [9]. Thus, Godlewski et al. introduced a switch for the dichotomy between a migrating and proliferating phenotype in glioblastoma that is also known as the* Go or Grow* principle [12].

Mathematical modelling has evolved as a useful tool for biology and medicine in gaining a better understanding of diseases and biological processes [13, 14]. Consequently, one application of mathematical models is the investigation of existing therapies and the development of new treatment options. This holds in particular true for models of cancer and cancer treatment [15–17]. In general, these models can be distinguished by the scale (macroscopic, microscopic or molecular) that they describe [14]. While on the macroscopic scale mainly general growth processes [18], deformation effects [19–21], or the response to radiotherapy [22–24] are examined, models on the microscopic scale deal among others with the interaction of tumour cells with their microenvironment (e.g., the immune system, the extracellular matrix, or nutrients) [25–28]. Molecular scale models on the contrary focus on effects of mutations and on molecular interaction and signalling pathways [29, 30]. By definition, all models constitute a simplification of reality. However, to obtain a more accurate model of reality, the coupling of models of different scales is unavoidable. In the case of cancer modelling, a few multiscale approaches exist [31]. In [32, 33], a model for glioblastoma growth was introduced that combines an agent based model with an EGFR (epidermal growth factor receptor) signalling network and focuses on the determination of the cell phenotypes “migrating” and “proliferating.” Later, also a model for the progression of lung cancer was developed [34] in that essentially only the molecular interaction network was interchanged. In [35], a model of cell-cell adhesion is presented that links intracellular E-cadherin signalling to a lattice-free model on the cellular scale.

All mathematical models have in common that they contain parameters (e.g., diffusion constants, radiosensitivity parameters, absorption, secretion, mutation, or kinetic reaction rates). These parameters might vary from patient to patient or even from cell to cell and need to be determined/estimated from experimental or patient data in the model development process [36]. One important problem is to examine the dependence of the modelled system on the model parameters, that is, to investigate how the system behaviour changes if one or more parameters are varied. This is realized by means of a sensitivity analysis [37, 38]. The goal of such an analysis is to identify those parameters that considerably influence the model, that is parameters are identified to that the system reacts sensitively. On the one hand it is important to estimate these parameters accurately, on the other hand these parameters indicate potential targets for new therapies [39]. So far, sensitivity analyses have been conducted nearly exclusively on a single scale; that is, the parameters that are varied and the system output that is measured belong to the same modelling scale [40]. This might be sufficient for a single scale model; however, for models covering more than one scale, such an analysis would cover only parts of the model and is therefore nonsatisfying. In [39, 40], an approach for a multiscale sensitivity analysis of a multiscale growth model for lung cancer is introduced that couples the molecular and cellular scale. For this analysis, parameters of the molecular scale part of the model are modified (in particular, they variegated the initial conditions for a system of ordinary differential equations (ODEs)). The dependence of the model on these parameters is evaluated at the cellular scale. Thus, the sensitivity analysis lives up to the multiscale modelling concept.

In [41], we presented a multiscale model of glioblastoma growth. On the cellular scale, an agent based model (ABM) represents individual cell actions and a partial differential equation (PDE) models the diffusion of a nutrient (glucose). Each cell is further equipped with a molecular interaction network that implements the regulation of cell migration and cell proliferation via the above described glucose-miR-451 signalling pathway. This interaction network is represented by a system of nine ODEs that describe the dependence of the nine relevant molecular species. In total, these nine equations contain 31 reaction parameters that were either taken from the literature or estimated to fit experimental data. We validated our multiscale model by comparing our* in silico* simulation results to* in vitro* experiments.

In the work at hand we analyse how the cellular scale of our model depends on the reaction parameters of the molecular interaction network. Motivated by the idea presented in [39, 40], parameters on the molecular scale are modified and the effect on the cellular scale is measured. In particular, we vary each of the 31 reaction parameters by multiplication with factors in the range [0.01,100] (i.e., also very extreme changes are considered) and consider different nutrient conditions. As a model output, we measure the total number of tumour cells and the number of migrating and of proliferating cells as a degree for the tumour volume and tumour build-up, respectively. Furthermore, the number of time steps necessary for a simulation to finish is evaluated as a measure for the expansion velocity. This allows for investigating which parameter changes have a significant impact on the model and, in particular, identifying the parameters and their respective changes that have a “positive” effect from the therapy development view.

In the following, we will first briefly recapitulate the major aspects of our multiscale model, before we introduce the method for the multiscale sensitivity analysis. Finally, we will present the results of the analysis and draw some conclusions for future research.

## 2. A Cross-Scale Model of Tumour Growth

To model the progression of GB in a region of a few square millimetres (to be exact 3 mm × 3 mm), a cross-scale model bridging the molecular and cellular scale has been previously developed [41, 42]. In this model, an intracellular molecular interaction network (represented by a system of ODEs) is coupled with an agent based model. Each agent represents a single tumour cell as is described below.

### 2.1. Molecular Interaction Model

**Figure 1:**
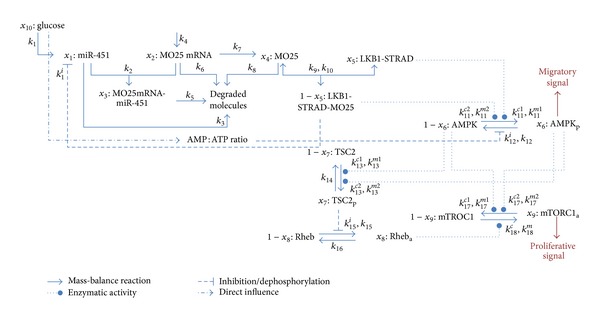
The molecular interaction network. Different arrow types pointing from one molecular species (given by the variable notation *x*_*i*_ and the biological term) to another indicate different kinds of reactions (see legend in figure for more details). The labels next to the reaction arrows denote the reaction parameters that are involved in the respective reactions. Adapted from [41]. Adapted with permission.

The molecular interaction model describes the influence of the extracellular glucose level on the proliferating versus migrating phenotype emergence of GB cells. The glucose level controls the concentration of miR-451 that in turn regulates the concentration of the active LKB1-MO25-STRAD complex [9]. This complex catalyses the phosphorylation of AMPK that influences cell proliferation via the mTORC1 pathway [10] and cell polarity through further signalling cascades [43]. The interaction model is depicted in Figure 1.

The molecular interactions are modelled by mass-balance reactions and Michaelis-Menten equations for the enzyme kinetics. In total, the concentration development of the nine molecular species is mathematically represented by a nondimensionalised system of nine nonlinear ODEs. These ODEs involve 31 reaction parameters which are given together with the details on the system of ODEs in [41]. The ODEs and the reaction parameters are also summarized in the appendix.

### 2.2. Cellular and Microenvironment Model

To model cellular processes (in terms of migration and proliferation) and the influence of environmental factors (in terms of the available nutrient concentration) on a tumour cell, an agent based modelling (ABM) approach is employed. In this model, each agent represents a single tumour cell.

Comparable to* in vitro* experiments in a petri dish, in this computational model, tumour growth is investigated in a two-dimensional region. The region of interest (3 mm × 3 mm) is represented by a regular grid of 200 × 200 grid points. Each of these grid points either can hold a tumour cell (the grid spacing of 1.5 × 10^1^ *μ*m × 1.5 × 10^1^ *μ*m corresponds to the average size of a GB cell [44]) or is empty.

Furthermore, each grid point holds information on the local nutrient level, in particular on the concentration of glucose. Initially, the glucose concentration (*x*_10_(0)) is assumed to be either constant or randomly distributed across the whole 3 mm × 3 mm region. Throughout the simulation, glucose is consumed by the tumour cells, cannot pass through the boundaries of the grid, and diffuses across the grid. The last is modelled by the use of a PDE of the form

(1) $$\begin{array}{c} \frac{\partial x_{10}}{\partial t}=\alpha D\nabla^{2}x_{10}, \end{array}$$

where *D* is the diffusion coefficient for glucose and *α* a factor that realizes slower diffusion in case a grid point is occupied by a tumour cell.

Tumour cells can migrate on the grid by moving to an empty neighbouring site or proliferate by placing a daughter cell on an empty neighbouring site. Migration and proliferation are governed by chemotaxis; that is, the movement is directed along a chemotactic gradient (here, glucose acts as the chemotactic agent).

### 2.3. Bridging the Modelling Scales

To incorporate information from the gene and protein scale into the cellular level, each cell/agent on the grid (as described in Section 2.2) is provided with a molecular interaction network (as given in Section 2.1). In each time step, each cell is provided with information on the local glucose level. Based on this information, the system of ODEs is evaluated independently for each individual cell. Then, the new phenotype of each cell (migrating, proliferating, or quiescent) is determined on basis of the concentration of AMPK (*x*_6_) and mTORC1 (*x*_9_). According to this phenotype, the cells are moved to a new location, a daughter cell is placed at a neighbouring site (after a certain delay to incorporate different time scales of migration and proliferation), or no interaction takes place.

**Figure 2:**
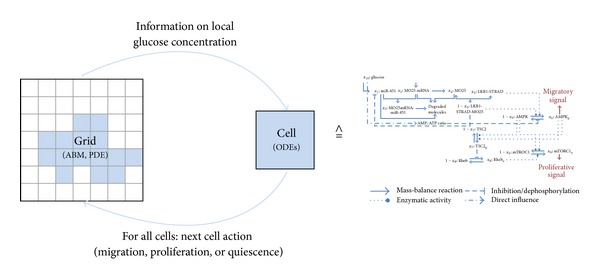
General model setup. The scheme shows how the different scales are combined. From the cellular grid on which the model is described by an ABM and a PDE, information about the local glucose concentration is transferred to each individual cell. Each cell is equipped with a system of ODEs describing the molecular interaction network. After simulating this system of ODEs, the cell's phenotype (migrating, proliferating, or quiescent) is determined. This phenotype then decides on the action of each individual cell on the cellular scale (i.e., the phenotype is realized on the grid).

**Figure 3:**
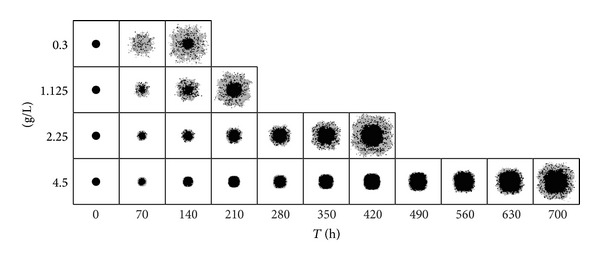
The spatiotemporal tumour development for four different initial glucose concentrations (from top to bottom: 3 × 10^−1^ gL^−1^, 1.125 gL^−1^, 2.25 gL^−1^, and 4.5 gL^−1^). Black denotes quiescent, dark gray migrating, and light gray proliferating tumour cells. Adapted from [41]. Adapted with permission.

The general model setup is depicted in Figure 2. Representative simulation results are shown in Figure 3. In these simulations, a constant initial glucose concentration *x*_10_(0)∈{3 × 10^−1^ gL^−1^, 1.125 gL^−1^, 2.25 gL^−1^, 4.5 gL^−1^} is assumed (a setting comparable to* in vitro* experiments). Initially, 797 cells are placed in a circular shape in the center of the grid. All simulations were run until the first cell reached the boundary. As could be seen in [41], these simulation results are in good agreement with results from* in vitro* experiments.

## 3. Sensitivity Analysis

In total, 31 parameters determine the behaviour of the tumour growth model by regulating nine ODEs. One important question one should consider is whether there exist one or more parameters that have a crucial influence on the whole system. In particular, when considering novel targets for therapeutic interventions, one searches for parameters that slow down the tumour expansion and decrease the tumour volume.

Sensitivity analyses are a tool to investigate the dependence of a system (e.g., a specific model outcome) on individual model parameters. The general procedure is to vary one or more parameters and measure the impact on the system. This allows identifying parameters that significantly influence the whole system. Generally, local sensitivity analyses (LSAs) and global sensitivity analyses (GSAs) can be distinguished [37]. In an LSA, all parameters are varied independently and, for each single variation, the impact on the system is measured. This allows for a rather simple and fast realization of the whole analysis. In contrast, a GSA implies that several parameter variations are combined (up to the exhaustion of all possible combinations) and the impact of these combined simultaneous perturbations is measured. This involves rather complex calculations and is computationally expensive. For the purpose of this study, it was sufficient to conduct an LSA and to further analyse the impact of the combined variation of two parameters at a time.

The general idea of this sensitivity analysis was to investigate the effects of changes in the intracellular setting of the tumour cells on the cell behaviour and overall tumour expansion. If, for example, a certain parameter significantly slowed down the tumour expansion, the respective reaction could be an indicator for future therapy targets. To accommodate for such an analysis across different spatial scales, we followed the idea introduced in [40]. The parameters were varied on the subcellular scale; that is, the reaction parameters of the system of ODEs that represents the molecular interaction network were altered. However, the system output was measured on the cellular level; that is, values that embody the tumour expansion were recorded. These values were then compared to the original standard setting to allow for an assessment of the influence of the parameter.

Initially, the simulation is performed with the original parameter setting and the model output *M* is recorded. Next, we multiplied each reaction parameter *k*_*l*_ (*k*_*l*_ ∈ {*k*_1_,…, *k*_18_^*m*^}) with different factors *b*_*n*_ ∈ *R*. For each of these combinations, *k*_*l*_^*b*_*n*_^ : = *k*_*l*_ · *b*_*n*_ of a parameter *k*_*l*_ and a factor *b*_*n*_ separate simulations are performed and the model output *M*_*k*_*l*_,*b*_*n*__ is recorded. For each parameter-factor setting, the simulation is run three times and the mean of the model output is calculated to account for the random elements of the model. Finally based on the model outputs and the parameter changes sensitivity coefficient *S*_*k*_*l*_,*b*_*n*__ are calculated as follows (see [37]):

(2) $$\begin{array}{c} S_{k_{l},b_{n}}:=\frac{\Delta M_{k_{l},b_{n}}/M}{\Delta k_{l}^{b_{n}}/k_{l}}=\frac{\left(M_{k_{l},b_{n}}-M\right)/M}{\left(k_{l}\cdot b_{n}-k_{l}\right)/k_{l}}=\frac{M_{k_{l},b_{n}}/M-1}{b_{n}-1} \end{array}$$

with Δ*M*_*k*_*l*_,*b*_*n*__ : = *M*_*k*_*l*_,*b*_*n*__ − *M* and Δ*k*_*l*_^*b*_*n*_^ = *k*_*l*_ · *b*_*n*_ − *k*_*l*_. These sensitivity coefficients facilitate the evaluation of the model outcome relative to the responsible parameter change.

Following this definition, the undermentioned observations can be made regarding the properties of the sensitivity coefficients *S*_*k*_*l*_,*b*_*n*__.

1. If *b*_*n*_ < 1 (i.e., the parameter *k*_*l*_ is decreased) and *M*_*k*_*l*_,*b*_*n*__ < *M* (i.e., the model output is decreased as well), then the respective sensitivity coefficient is positive (i.e., *S*_*k*_*l*_,*b*_*n*__ > 0). The same holds true if *b*_*n*_ > 1 and *M*_*k*_*l*_,*b*_*n*__ > *M*. If, on the other hand, *b*_*n*_ < 1 and *M*_*k*_*l*_,*b*_*n*__ > *M* or *b*_*n*_ > 1 and *M*_*k*_*l*_,*b*_*n*__ < *M*, this results in a negative sensitivity coefficient (i.e., *S*_*k*_*l*_,*b*_*n*__ < 0).
2. If the same parameter *k*_*l*_ is separately multiplied with two different factors *b*_1_ and *b*_2_ for which *b*_1_ − 1 = 1 − *b*_2_ and if additionally the resulting outcomes *M*_*k*_*l*_,*b*_1__ and *M*_*k*_*l*_,*b*_2__ do not differ (i.e., *M*_*k*_*l*_,*b*_1__ = *M*_*k*_*l*_,*b*_2__), then the resulting sensitivity coefficients are the same except for the sign (i.e., *S*_*k*_*l*_,*b*_1__ = −*S*_*k*_*l*_,*b*_2__).
3. If the same parameter *k*_*l*_ is separately multiplied with two different factors *b*_1_ and *b*_2_ that result in the same simulation outcome *M*_*k*_*l*_,*b*_1__ = *M*_*k*_*l*_,*b*_2__, then the smaller factor results in the larger respective sensitivity coefficient (i.e., *b*_1_ < *b*_2_⇒*S*_*k*_*l*_,*b*_1__ > *S*_*k*_*l*_,*b*_2__ and vice versa).

For the model output *M*_*k*_*l*_,*b*_*n*__^*e*^, we choose four different endpoints *e* that are representative for the respective tumour expansions and tumour characteristics. As a measure for the tumour expansion velocity, we recorded the number of simulation time steps *M*_*k*_*l*_,*b*_*n*__^time^ necessary for the first tumour cell to reach the boundary of the simulation region. A high expansion velocity corresponds to a short time until the first cell reaches the boundary (i.e., a small *M*_*k*_*l*_,*b*_*n*__^time^) and vice versa. The final tumour volume is estimated by the number of cells in the last time step *M*_*k*_*l*_,*b*_*n*__^total^. A large tumour volume is associated with a large number of tumour cells and a small volume with a small number of tumour cells. To further get a better understanding of the tumour build-up, additionally, the number of migrating cells in the last time step *M*_*k*_*l*_,*b*_*n*__^mig^ and the number of proliferating cells in the last time step *M*_*k*_*l*_,*b*_*n*__^prolif^ were documented. For all these four model outputs *M*_*k*_*l*_,*b*_*n*__^*e*^, the corresponding sensitivity coefficients were calculated and were named accordingly *S*_*k*_*l*_,*b*_*n*__^time^, *S*_*k*_*l*_,*b*_*n*__^total^, *S*_*k*_*l*_,*b*_*n*__^mig^, and *S*_*k*_*l*_,*b*_*n*__^prolif^.

This setup allows for the assessment of the effects of changes in the reaction parameters on the overall behaviour of the tumour (tumour expansion and tumour build-up) as well in absolute as in relative terms.

## 4. Results

The model is implemented in C++. For the sensitivity analysis, each of the 31 reaction parameters of the molecular interaction model was separately multiplied with the factors 0.01, 0.1, 0.5, 0.8, 0.9, 0.95, 0.98, 0.99, 1.01, 1.02, 1.05, 1.1, 1.2, 1.5, 1.9, 1.99, 5, 10, 50, and 100.

As could be seen in Figure 3, the tumour growth behaves very different in different glucose environments. Therefore, the sensitivity analysis was also carried out for tumours growing under different initial glucose conditions *x*_10_(0). In particular, for each parameter-factor combination, the tumour growth simulation was run three times for each of the four initial glucose settings (3 × 10^−1^ gL^−1^, 1.125 gL^−1^, 2.25 gL^−1^, and 4.5 gL^−1^). Thus, in total, simulations for 2480 different settings were carried out.

Each simulation was run until the first cell reached the boundary, the necessary number of time steps *M*_*k*_*l*_,*b*_*n*__^time^(*x*_10_(0)), the final number of all tumour cells *M*_*k*_*l*_,*b*_*n*__^total^(*x*_10_(0)), and the number of migrating *M*_*k*_*l*_,*b*_*n*__^mig^(*x*_10_(0)) and proliferating *M*_*k*_*l*_,*b*_*n*__^prolif^(*x*_10_(0)) cells in the last time step were recorded, and the respective sensitivity coefficients *S*_*k*_*l*_,*b*_*n*__^*e*^(*x*_10_(0)) were calculated for all four endpoints *e*. For the ease of notation, the reference to the parameter *k*_*l*_, the factor *b*_*n*_, and the initial glucose concentration *x*_10_(0) will be omitted in the following, if the sensitivity coefficients are discussed in general; that is, they will be referred to as *S* ^*e*^.

### 4.1. Sensitivity Coefficients

**Figure 4:**
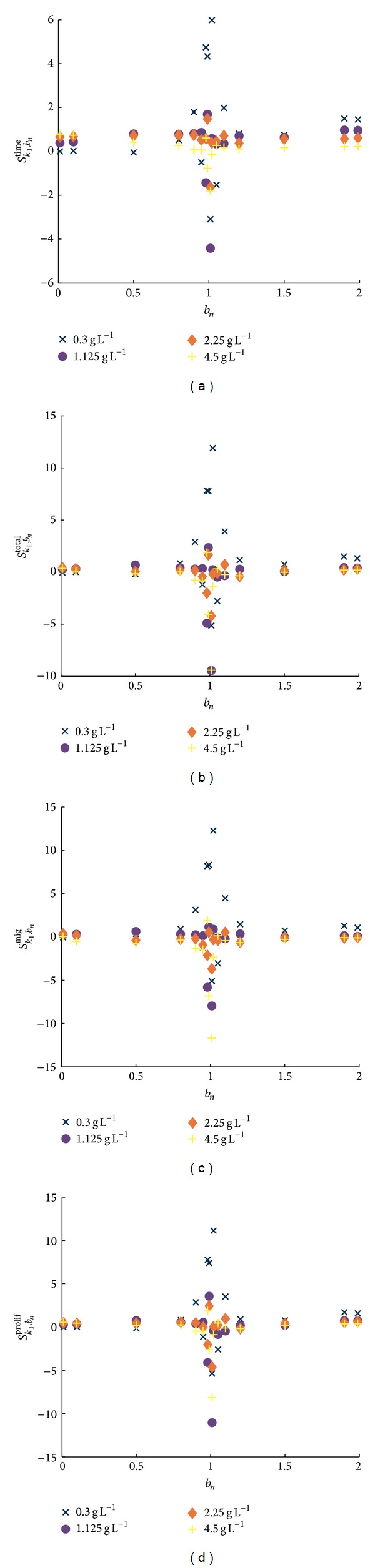
The sensitivity coefficients for parameter *k*_1_ for the tested factors *b*_*n*_ in the interval ]0; 2[ for all four tested initial glucose levels 3 × 10^−1^ gL^−1^, 1.125 gL^−1^, 2.25 gL^−1^, and 4.5 gL^−1^. (a) The sensitivity coefficients for the number of time steps necessary for the first cell to reach the boundary, (b) the sensitivity coefficients for the total number of cells in this last time step, (c) the sensitivity coefficients for the number of migrating cells, and (d) the sensitivity coefficient for the number of proliferating cells, respectively. Each marker represents the mean result of three simulations with the same parameter scaling with the same initial level of glucose. In all these figures, the black crosses (×) represent the data for an initial glucose level of 3 × 10^−1^ gL^−1^, the blue circles (●) for an initial glucose concentration of 1.125 gL^−1^, and the red diamond (◆) and the yellow plus sign (+) for an initial glucose level of 2.25 gL^−1^ and 4.5 gL^−1^, respectively.

Here, only the results of the sensitivity coefficient calculations for one parameter (*k*_1_) are shown in detail (see Figure 4). Results for the other parameters are summarized in Table 1 and in the electronic supplementary material (see Figures S2–S13 in Supplementary Material available online at http://dx.doi.org/10.1155/2014/437094).

In Figure 4, the sensitivity coefficients for the four glucose settings are shown for the parameter *k*_1_. The range of the factors shown in Figure 4 is limited to factors *b*_*n*_ < 2. By this means, an equal number of factors are shown that decrease and increase the original parameter *k*_1_ by the same intervals, respectively. Furthermore, multiplication of the original parameter with a high factor (5, 10, 50, and 100) tends to result in rather low sensitivity coefficients (compare the definition of *S*_*k*_*l*_,*b*_*n*__ and the observations made in Section 3).

Figure 4(a) shows the relative change of the number of time steps necessary to finish a simulation. In Figure 4(b), the sensitivity coefficients for the total number of tumour cells in the last time step can be seen. Figures 4(c) and 4(d) display the sensitivity coefficients for the number of migrating and proliferating cells in the last time step of the simulation, respectively. In all these figures, the crosses (×) represent the data for an initial glucose level of 3 × 10^−1^ gL^−1^, the circles (●) for an initial glucose concentration of 1.125 gL^−1^, and the diamond (◆) and the plus sign (+) for an initial glucose level of 2.25 gL^−1^ and 4.5 gL^−1^, respectively.

For all of the four initial glucose settings, factors close to one cause the largest deviation of the four sensitivity coefficients *S*_*k*_1_,*b*_*n*__^*e*^ from zero. However, if the parameter *k*_1_ is multiplied with factors *b*_*n*_ close to zero or two, this results in low sensitivity coefficients. The absolute maximum value of all sensitivity coefficients *S*_*k*_1_,*b*_*n*__^*e*^

(3) $$\begin{array}{c} m_{k_{1}}^{e}=\underset{b_{n},x_{10}(0)}{\max}\left|S_{k_{1},b_{n}}^{e}\left(x_{10}\left(0\right)\right)\right| \end{array}$$

is reached for a low initial glucose concentration of 3 × 10^−1^ gL^−1^. With an increasing initial glucose level, the amplitudes of the sensitivity coefficients tend to decrease.

The graphs of the sensitivity coefficients for the other parameters look similar to the ones explained above. The main differences affect the range of the sensitivity coefficients and the initial glucose concentration for which the maximum *m*_*k*_*l*__^*e*^ is taken. Therefore, Table 1 provides the maximal absolute sensitivity coefficients *m*_*k*_*l*__^*e*^ together with the respective glucose values *x*_10_(0) that result in these maximal values for all parameters. In contrast to Figure 4, all factors from 0.01 to 100 were explored. The complete results for all reaction parameters and all glucose settings are given in the electronic supplementary material (Figures S2–S13).

From the table, it can be seen that, for all sensitivity coefficients *S*_*k*_*l*_,*b*_*n*__^*e*^, the maxima of the absolute values *m*_*k*_*l*__^*e*^ are primarily reached for the lowest glucose level of *x*_10_(0) = 3 × 10^−1^ gL^−1^ for all parameters. Only a few parameters exist for the absolute maximum *m*_*k*_*l*__^*e*^ for a medium glucose level (1.125 gL^−1^ or 2.25 gL^−1^). The absolute maxima vary between 3.71–17.94 and 8.52–38.42 for *S* ^time^ and *S* ^total^, respectively. For *S* ^mig^, the absolute maxima are in the range 9.44–43.66 and the sensitivity coefficients for the number of proliferating cells in the last time step *S* ^prolif^ take maximum values between 8.54 and 34.89. The maximum over all parameters of all maximal sensitivity coefficients *m*_*k*_*l*__^*e*^ (max⁡_*k*_*l*__(*m*_*k*_*l*__^*e*^)) is taken for the parameter *k*_4_. Except for the endpoint *e* = time, the parameter *k*_17_^*m*2^ results in the lowest *m*_*k*_*l*__^*e*^.

### 4.2. Correlation between Changes in *M* ^time^ and *M* ^total^

Besides investigating the sensitivity coefficients *S* ^time^, *S* ^total^, *S* ^mig^, and *S* ^prolif^, we also analysed the correlation between changes in *M* ^time^ and *M* ^total^. Thus, we examined the correlation between tumour expansion velocity and tumour volume for different parameter variations. This is motivated by the fact that the number of time steps necessary for the first tumour cell to reach the boundary *M* ^time^ can be considered as a measure for the velocity of the tumour expansion and the total number of tumour cells in the last time step *M* ^total^ is a direct measure for the tumour volume.

We define

(4) $$\begin{array}{c} \delta M_{k_{l},b_{n}}^{j}:=\frac{\Delta M_{k_{l},b_{n}}^{j}}{M^{j}}=\frac{M_{k_{l},b_{n}}^{j}}{M^{j}}-1,\;j\in \left\{\text{time},\text{total}\right\} \end{array}$$

with Δ*M*_*k*_*l*_,*b*_*n*__^*j*^ and *M* as in Section 3. If the total number of cells decreases for a certain parameter variation, we can observe that this corresponds to *δM*_*k*_*l*_,*b*_*n*__^total^ < 0. Furthermore, if the variation of parameter *k*_*l*_ by multiplication with a factor *b*_*n*_ causes an increase in the number of time steps necessary for a simulation to terminate, this comes along with *δM*_*k*_*l*_,*b*_*n*__^time^ > 0. In general, *δM*_*k*_*l*_,*b*_*n*__^*j*^ = *a* can be translated into *M*_*k*_*l*_,*b*_*n*__^*j*^ = (*a* + 1) · *M*.

**Figure 5:**
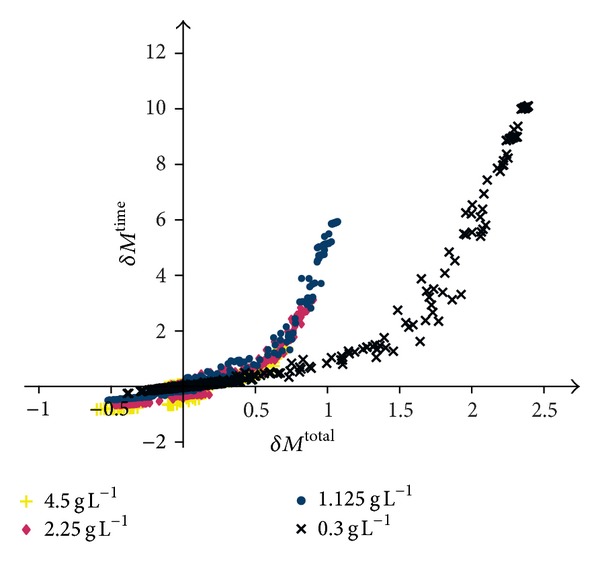
The correlation between the changes in the number of time steps necessary for a simulation to complete and the number of tumour cells in this last time step for all tested parameter scalings. Each marker represents the mean result of three simulations with the same parameter scaling and with the same initial level of glucose. The black crosses (×) represent the data for an initial glucose level of 3 × 10^−1^ gL^−1^, the blue circles (●) for an initial glucose concentration of 1.125 gL^−1^, and the red diamond (◆) and the yellow plus sign (+) for an initial glucose level of 2.25 gL^−1^ and 4.5 gL^−1^, respectively.

Figure 5 shows the correlation of the number of time steps and the number of tumour cells with *δM*_*k*_*l*_,*b*_*n*__^total^ on the *x*-axis and *δM*_*k*_*l*_,*b*_*n*__^time^ on the *y*-axis. Each marker corresponds to a simulation with a different parameter scaling. As before, the simulations were run for four different glucose settings and the crosses (×, in black) represent the data for an initial glucose level of 3 × 10^−1^ gL^−1^, the circles (●, in blue) for an initial glucose concentration of 1.125 gL^−1^, and the diamond (◆, in red) and the plus sign (+, in yellow) for an initial glucose level of 2.25 gL^−1^ and 4.5 gL^−1^, respectively.

For each glucose setting, an increase in the number of time steps comes along with an increase in the number of tumour cells. A decrease of *M* ^time^ corresponds to a decrease of *M* ^total^. The increases are most prominent for a low initial glucose level of 3 × 10^−1^ gL^−1^. For medium glucose levels (1.125 gL^−1^ and 2.25 gL^−1^), one mainly observes an increase in the number of time steps (for a slight increase of *M* ^total^) and a decrease of the number of tumour cells (along with a slight decrease of *M* ^time^). Assuming a high glucose level (4.5 gL^−1^), primarily a decrease of the number of time steps and a decrease of the final total number of tumour cells become apparent. None of the parameter scalings results in a significant decrease of the final total number of tumour cells at the same time with an increase in the number of time steps.

### 4.3. Combined Parameter Changes

The goal of therapies should be to reduce the tumour expansion velocity and to reduce the tumour volume at the same time (i.e., in Figure 5, it would be desirable to end up in the upper left quadrant). Therefore, we identified the parameter-factor combinations whose corresponding simulations resulted in *δM* ^time^ > 9 for an initial glucose value of 3 × 10^−1^ gL^−1^ (i.e., the marker group in the upper right of Figure 5). Furthermore, we determined the parameter-factor combinations that result in simulations with *δM* ^time^ < −0.8 assuming an initial glucose level of 4.5 gL^−1^. The goal is to combine two of these different variations at a time to allow for a semiglobal sensitivity analysis.

The respective parameters and factors are given in Table 2. The column Factor+ includes those factors whose multiplication with a given parameter resulted in *δM* ^time^ > 9 (for 3 × 10^−1^ gL^−1^ glucose concentration), and Factor− refers to those factors whose multiplication with a given parameter resulted in *δM* ^total^ < −0.8 (for 4.5 gL^−1^ glucose concentration). It can be noted that Table 2 lists 14 out of the 31 reaction parameters. The variation of 9 out of these 14 parameters resulted in *δM* ^time^ > 9 (for 3 × 10^−1^ gL^−1^ glucose concentration) and *δM* ^total^ < −0.8 (for 4.5 gL^−1^ glucose concentration) by multiplication with different factors. Out of all 20 factors (from 0.01 to 100), the highest and lowest scaling factors (0.01, 0.1, 10, 50, and 100) predominantly resulted in a high number of time steps for a low glucose value or a low total number of cells for a high glucose value. No factors close to one appear in Table 2.

**Figure 6:**
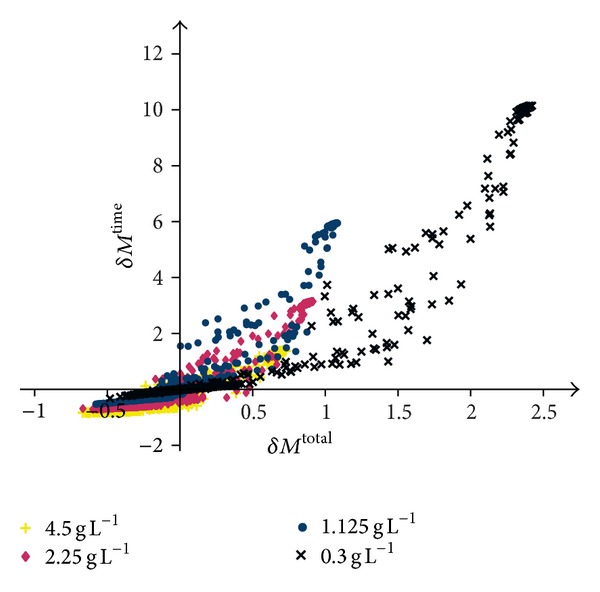
The correlation between the changes in the number of time steps necessary for a simulation to complete and the number of tumour cells in this last time step for all tested combined parameter scalings. Each marker represents the mean result of three simulations with the same combination of parameter scalings with the same initial level of glucose. The black crosses (×) represent the data for an initial glucose level of 3 × 10^−1^ gL^−1^, the blue circles (●) for an initial glucose concentration of 1.125 gL^−1^, and the red diamond (◆) and the yellow plus sign (+) for an initial glucose level of 2.25 gL^−1^ and 4.5 gL^−1^, respectively.

Next, we explore all possible combinations of two parameter-factor pairs (that are listed in Table 2) at a time; that is, simulations are run in which two parameters *k*_*l*1_, *k*_*l*2_ are modified: one parameter (*k*_*l*1_) with an associated factor (*b*_*n*1_) from the Factor+ group and another parameter (*k*_*l*2_) with a corresponding factor (*b*_*n*2_) out of the Factor− group. Obviously, there are some parameter-factor pairs that cannot be combined, since, for example, it is impossible to vary parameter *k*_1_ at the same time by the factors 10 and 0.01. In total, 603 combinations are possible and, for each of these combinations, three simulations are carried out for each of the four glucose settings. For each of the simulations, the necessary number of time steps for the first cell to reach the boundary and the total number of cells in the last time step are evaluated and *δM* ^time^ and *δM* ^total^ are calculated based on the mean values. The results are shown in Figure 6 in the same manner as the correlation between *M* ^time^ and *M* ^total^ was previously presented in Figure 5 for just one parameter change.

For a low initial glucose concentration (3 × 10^−1^ gL^−1^), most variations resulted in a significant increase of the number of time steps (*δM* ^time^ > 0) and of the number of tumour cells (*δM* ^total^ > 0). For the medium and high glucose levels (1.125 gL^−1^, 2.25 gL^−1^, and 4.5 gL^−1^), some combined parameter variations exist that yield a positive *δM* ^time^ > 0 and at the same time a negative *δM* ^total^ < 0. These combined parameter variations, therefore, cause a decrease of the tumour expansion velocity and of the tumour volume. For the low initial glucose concentration (3 × 10^−1^ gL^−1^), no such combination of parameter changes exists.

For initial glucose levels of 1.125 gL^−1^, 2.25 gL^−1^, and 4.5 gL^−1^, Table 3 lists the combinations of parameter scalings (*k*_*l*1_ · *b*_*n*1_ combined with *k*_*l*2_ · *b*_*n*2_) that resulted in an increase of the number of time steps (*δM* ^time^ > 0.01) along with a decrease of the total number of tumour cells (*δM* ^total^ < −0.01). Instead of using exactly zero as the threshold, an additional margin of 0.01 is applied. Thus, combinations are excluded that influence the system output only very slightly.

A few combined parameter scalings result in a decrease of the tumour expansion velocity and the tumour volume for all medium and high glucose levels (1.125 gL^−1^, 2.25 gL^−1^, and 4.5 gL^−1^), in particular, as follows:

- *k*_2_ · 50 combined with *k*_9_ · 100 or *k*_10_ · 0.01,
- *k*_11_^*c*2^ · 50 combined with *k*_8_ · 100.

On the one hand, some of the parameter scalings caused a decrease of the tumour expansion velocity and the tumour volume only in combination with a few (1–3) other parameter modifications across all initial glucose concentrations. On the other hand, four parameter scalings (*k*_1_ · 50, *k*_2_ · 50, *k*_4_ · 50, and *k*_11_^*c*2^ · 50) could be combined with a quite large selection of other parameter variations (≥5 combinations across all initial glucose settings).

## 5. Discussion and Conclusion

Molecular targeted therapies are theoretically a promising approach for glioblastoma treatment. However, so far, none of the existing agents could significantly improve the overall survival [2]. Therefore, further research is inevitable: already well-known signaling networks as well as newly discovered ones need to be examined further for their therapeutic potential. For this purpose, mathematical modelling is a useful tool. In the work at hand, we have presented an approach for identifying influential parameters in a cross-scale model of glioblastoma growth. This previously developed model [41] describes the relevant cellular processes (migration and proliferation) in combination with an intracellular molecular interaction network. A multiscale sensitivity analysis allows us to measure the effect on the tumour growth if reaction parameters on the molecular scale are perturbed. Parameter variations that “positively” influence the tumour progression (i.e., slower expansion velocity and smaller tumour volume) indicate potential targets for new therapeutic interventions. So far, only sensitivity analyses that cover processes on a single scale or that solely variegate the initial conditions for a lung cancer model have been presented [39, 40]. To our knowledge, here, we have presented for the first time a multiscale sensitivity analysis for a model of glioblastoma growth that not only accounts for a systematic variation of all reaction parameters but also considers different nutrient conditions. We could restrict the analysis to an LSA since for therapeutic purposes it is impossible to modify too many parts of a signaling network.

Godlewski et al. [9] discovered that microRNA-451 plays an essential role for the nutrient level depending decision of glioma cells to either migrate or proliferate. This miR-451 directly regulates the expression level of the protein MO25 that is relevant for further downstream signaling. Therefore, it was to be expected that those parameters that control the miR-451 and MO25 concentration will have a significant influence on the whole system. Indeed, Table 1 supports this presumption. For all four sensitivity coefficients under examination (*S* ^time^ as a measure for the expansion velocity, *S* ^total^ as a measure for the tumour volume, and *S* ^mig^ and *S* ^prolif^ as indicators for the build-up of the tumour), the maximum values among all parameter variations are taken for parameters that are relevant for the control of the level of miR-451 and MO25 (*k*_1_, *k*_2_, and *k*_4_).

In general, it can be observed (see Figure 4 and Figures S2–S13) that, measured in relative terms (cf. the definition of *S*_*k*_*l*_,*b*_*n*__, (2)), small parameter perturbations cause the largest changes for all parameters. For the same parameter-factor combination as well positive as negative sensitivity coefficients can be taken, depending on the initial glucose concentration. Furthermore, since the graphs for all parameters have a very similar structure (no parameter sets itself noticeably apart from the others), further conclusions on the effects of a parameter variation by means of these plots are hardly possible.

Nevertheless, on the basis of Table 1, it can be observed that the sensitivity coefficients primarily take their absolute maximum values (maximum over all 20 factors and 4 initial glucose values) for a low initial glucose concentration (3 × 10^−1^ gL^−1^). Since tumours growing in a low glucose environment expand very fast (which corresponds to a small number of time steps until the first cell reaches the boundary), any changes that only slightly slow down or speed up the expansion are considerably noticeable. The same holds true for the dependence of the tumour volume (corresponding to the number of tumour cells) on the parameter variations.

If one does not consider the relative but the absolute changes in the model output (cf. Figure 5), the first general observation is that an increase of the number of time steps (*δM* ^time^ > 0) goes along with an increase of the total number of tumour cells (*δM* ^total^ > 0) and vice versa. An increase of the number of tumour cells by a parameter variation is ultimately caused by an increase of the number of proliferating cells. Proliferation, in turn, is a process that happens much slower than migration and therefore the tumour expansion is slowed down. Thus, this relationship was to be expected in a more or less explicit form. The extreme changes for a low initial glucose condition (3 × 10^−1^ gL^−1^) are due to the fact that, in the original setting, the tumour expands very fast and is only very loosely packed, that is, consisting of rather few cells. Therefore, the potential for an increase of the number of time steps and the total number of tumour cells due to an increase of the number of proliferating cells is very high. Consequently, *δM* ^time^ and *δM* ^total^ reach high values. On the contrary, under a high glucose level (4.5 gL^−1^), the number of time steps and tumour cells is already rather high and it is hardly possible to increase these. However, also, the decrease due to the parameter variations turns out to be only modest. Unfortunately, none of the parameter perturbations resulted in a slower growing tumour that consists of fewer cells (no markers in the upper left quadrant of Figure 5) for any of the glucose settings. Thus, from a single parameter variation, no indicator for a potential therapy could be identified.

The identification of those parameters that resulted in the most extreme absolute changes (*δM* ^time^ < −0.8 and *δM* ^time^ > 9) of the model output (Table 2) yielded mainly parameters that control the “early” parts of the network. That is, primarily, reactions that involve miR-451 and MO25 cause these high variations. Furthermore, it can be noticed that only the multiplication of these parameters with very large or very small factors did change the tumour volume and expansion velocity considerably. Thus, in relative terms, small perturbations of the parameters cause large changes. However, in absolute terms, large parameter modifications are responsible for the large changes.

The combined scaling of two selected parameters (see Table 2) resulted in an overall similar relationship of the number of time steps and the total number of tumour cells (see Figure 6) as for a single parameter variation: an increase of *M* ^time^ coincides with an increase of *M* ^total^. However, a few pairs of parameter scalings could be identified that, for a medium to high glucose level, cause a less aggressive tumour expansion in the sense that the expansion velocity could be slowed down and the tumour volume could be decreased (*δM* ^time^ > 0.01 and *δM* ^total^ < −0.01). For a low initial glucose condition (3 × 10^−1^ gl^−1^), none of the variations resulted in the desirable effect. Table 3 further supports the key role of the reactions that regulate the miR-451 and MO25 level for a less aggressive tumour expansion. Most combinations listed in this table contain at least one parameter that controls the reactions that involve either miR-451 or MO25.

### 5.1. Conclusion

For a previously developed multiscale model of glioblastoma growth, we could identify a few combinations of reaction parameter modifications that result in a less aggressive tumour progression. Therefore, these parameters can also be thought of as potential targets for therapies. The parameters are primarily part of reactions that involve the microRNA-451 and the thereof dependent protein MO25 that are responsible for the further downstream signalling. Thus, the analysis supports the relevance of these two molecules for future therapy research.

## Supplementary Material

The supplementary material provides the complete results of the sensitivity coefficient calculations for all model parameters. For each parameter modification and each of the four system endpoints the corresponding sensitivity coefficients are presented in form of figures comparable to Figure 4 of the article.

## Figures and Tables

**Table 1:** The maximal absolute sensitivity coefficients. This table summarizes for each parameter *k*_*l*_ the maximum of the absolute values (*m*_*k*_*l*__^*e*^) of the four different sensitivity coefficients (*S* ^time^, *S* ^total^, *S* ^mig^, and *S* ^prolif^) among all factors *b*_*n*_ and all tested initial glucose values (*x*_10_(0)). The max⁡(∣·∣) values are dimensionless (d.u.) and the initial glucose concentration values *x*_10_(0) are given in g L^−1^.

*k*_*l*_	*S* ^time^		*S* ^total^		*S* ^mig^		*S* ^prolif^	
	*m*_*k*_*l*__^time^	*x*_10_(0)	*m*_*k*_*l*__^total^	*x*_10_(0)	*m*_*k*_*l*__^mig^	*x*_10_(0)	*m*_*k*_*l*__^prolif^	*x*_10_(0)
	d.u.	[g L^−1^]	d.u.	[g L^−1^]	d.u.	[g L^−1^]	d.u.	[g L^−1^]
*k*_1_	5.98	0.3	11.91	0.3	12.28	0.3	11.13	0.3
*k*_1_^*i*^	6.60	0.3	14.88	0.3	17.38	0.3	13.06	0.3
*k*_2_	13.20	0.3	21.69	0.3	22.99	0.3	20.78	0.3
*k*_3_	11.75	0.3	21.53	0.3	22.49	0.3	21.61	0.3
*k*_4_	17.94	0.3	38.42	0.3	43.66	0.3	34.89	0.3
*k*_5_	6.80	0.3	13.55	0.3	16.12	0.3	11.37	0.3
*k*_6_	6.80	0.3	9.88	0.3	10.55	0.3	9.56	0.3
*k*_7_	11.18	0.3	15.95	1.125	17.10	1.125	15.08	1.125
*k*_8_	5.57	0.3	13.63	2.25	15.01	2.25	12.92	2.25
*k*_9_	11.14	0.3	11.14	2.25	12.16	2.25	10.59	2.25
*k*_10_	8.25	0.3	13.76	0.3	14.77	0.3	13.16	0.3
*k*_11_^*m*1^	5.58	1.125	12.54	1.125	13.45	1.125	11.71	1.125
*k*_11_^*c*1^	3.71	0.3	11.33	2.25	11.95	2.25	10.95	2.25
*k*_11_^*m*2^	7.73	0.3	14.87	0.3	16.39	0.3	14.91	0.3
*k*_11_^*c*2^	12.43	0.3	14.21	0.3	16.55	0.3	12.31	0.3
*k*_12_^*i*^	8.66	0.3	18.44	0.3	20.67	0.3	16.93	0.3
*k*_12_	8.25	0.3	15.22	0.3	17.52	0.3	13.79	0.3
*k*_13_^*m*1^	10.93	0.3	21.62	0.3	23.88	0.3	19.88	0.3
*k*_13_^*c*1^	5.36	0.3	10.57	0.3	10.65	0.3	10.56	0.3
*k*_13_^*m*2^	11.96	0.3	19.50	0.3	19.63	0.3	19.31	0.3
*k*_13_^*c*2^	7.01	1.125	15.41	0.3	15.51	0.3	15.46	0.3
*k*_14_	8.66	0.3	18.14	0.3	21.16	0.3	14.95	0.3
*k*_15_^*i*^	6.49	1.125	12.11	1.125	10.14	1.125	14.23	1.125
*k*_15_	14.85	0.3	32.23	0.3	36.54	0.3	29.02	0.3
*k*_16_	9.04	0.3	15.40	0.3	17.48	0.3	13.18	0.3
*k*_17_^*m*1^	7.01	0.3	13.86	0.3	15.18	0.3	12.85	0.3
*k*_17_^*c*1^	6.19	0.3	12.30	1.125	13.24	1.125	10.97	1.125
*k*_17_^*m*2^	3.92	0.3	8.52	0.3	9.44	0.3	8.54	1.125
*k*_17_^*c*2^	9.95	0.3	20.23	1.125	22.69	1.125	18.10	1.125
*k*_18_^*m*^	9.90	0.3	11.81	0.3	11.73	0.3	11.71	0.3
*k*_18_^*c*^	6.80	0.3	14.18	0.3	15.80	0.3	12.30	0.3

**Table 2:** Parameters and corresponding scaling factors that result in a high number of time steps for a low glucose value (Factor+) or a low number of total cells for a high glucose value (Factor−).

Parameter	Factor+	Factor−
*k*_1_	10, 50, 100	0.01
*k*_1_^*i*^	—	0.01
*k*_2_	10, 50, 100	0.01
*k*_3_	0.01, 0.1	50, 100
*k*_4_	0.01, 0.1	50, 100
*k*_7_	0.01, 0.1	50, 100
*k*_8_	10, 50, 100	—
*k*_9_	0.01, 0.1	50, 100
*k*_10_	10, 50, 100	0.01
*k*_11_^*c*1^	—	10, 50, 100
*k*_11_^*m*2^	50, 100	—
*k*_11_^*c*2^	0.01, 0.1	5, 10, 50, 100
*k*_12_^*i*^	—	0.01, 0.1
*k*_12_	5, 10, 50, 100	0.01, 0.1

**Table 3:** The parameter scalings whose combinations result in a decrease of the tumour expansion velocity and the tumour volume. The first and second column list the parameters (*k*_*l*1_) and factors (*b*_*n*1_) whose corresponding variations (*k*_*l*1_ · *b*_*n*1_) combined with the second parameter scalings (*k*_*l*2_ · *b*_*n*2_) (given for different initial glucose concentrations in the third (1.125 gL^−1^), fourth (2.25 gL^−1^), and fifth (4.5 gL^−1^) column) resulted in *δM* ^time^ > 0.01 and *δM* ^total^ < −0.01.

*k*_*l*1_	*b*_*n*1_	*k*_*l*2_∗*b*_*n*2_		
		1.125 g L^−1^	2.25 g L^−1^	4.5 g L^−1^
*k*_1_	0.01	—	—	*k*_7_ · 100
	10	—	*k*_11_^*c*2^ · 5	—
	50	*k*_10_ · 0.01	*k*_9_ · 100; *k*_11_^*c*2^ · 10	*k*_1_^*i*^ · 0.01; *k*_9_ · {50,100}; *k*_10_ · 0.01

*k*_1_^*i*^	0.01	—	*k*_8_ · 50	*k*_8_ · 50

*k*_2_	0.01	—	—	*k*_4_ · 0.01; *k*_7_ · 0.01
	50	*k*_1_^*i*^ · 0.01; *k*_9_ · 100; *k*_10_ · 0.01	*k*_1_^*i*^ · 0.01; *k*_9_ · 100; *k*_10_ · 0.01	*k*_9_ · 100; *k*_10_ · 0.01; *k*_11_^*c*2^ · 10

*k*_4_	50	*k*_2_ · 100; *k*_3_ · 0.01; *k*_10_ · 100	*k*_9_ · 0.01	*k*_8_ · 100

*k*_7_	50	—	—	*k*_8_ · 100; *k*_9_ · 0.01

*k*_11_^*c*2^	5	*k*_8_ · 10; *k*_9_ · 0.1;	*k*_3_ · 0.01; *k*_4_ · 0.1;	*k*_8_ · 10; *k*_9_ · 0.1
		*k*_10_ · 10; *k*_12_ · 5	*k*_7_ · 0.1; *k*_10_ · 50	
	10	*k*_12_ · 10	—	—
	50	*k*_8_ · 100; *k*_9_ · 0.01	*k*_8_ · 100; *k*_12_ · 50	*k*_8_ · 100; *k*_9_ · 0.01; *k*_10_ · 100; *k*_12_ · 50
	100	—	*k*_12_ · 100	—

**Table 4:** Molecular species, their variable names, and units.

Species	Name	Unit
*x*_1_	miR451	pmol L^−1^
*x*_2_	MO25 mRNA	pmol L^−1^
*x*_3_	MO25 miRNA bound	pmol L^−1^
*x*_4_	MO25	pmol L^−1^
*x*_5_	LKB1-STRAD	pmol L^−1^
*x*_6_	AMPK phosphorylated	pmol L^−1^
*x*_7_	TSC2 phosphorylated	pmol L^−1^
*x*_8_	Rheb active	pmol L^−1^
*x*_9_	mTOR C1 active	pmol L^−1^
*x*_10_	Glucose	g L^−1^

**Table 5:** The reaction parameters (DC: dimensionless constant).

Parameter	Value	Unit
*k*_1_	1.2 × 10^−9^	pmol^−1^ L s^−1^
*k*_1_^*i*^	1	pmol L^−1^
*k*_2_	3 × 10^−2^	pmol^−1^ L s^−1^
*k*_3_	3 × 10^1^	s^−1^
*k*_4_	1.4583 × 10^−2^	s^−1^
*k*_5_	3.6 × 10^2^	s^−1^
*k*_6_	1.0467 × 10^−2^	s^−1^
*k*_7_	5.626 × 10^−3^	s^−1^
*k*_8_	6.4167 × 10^−3^	s^−1^
*k*_9_	6 × 10^−2^	pmol^−1^ L s^−1^
*k*_10_	3.6 × 10^2^	s^−1^
*k*_11_^*m*1^	5 × 10^2^	pmol L^−1^
*k*_11_^*c*1^	1.8 × 10^5^	pmol L^−1^ s^−1^
*k*_11_^*m*2^	5 × 10^2^	pmol L^−1^
*k*_11_^*c*2^	1.8 × 10^6^	pmol L s^−1^
*k*_12_^*i*^	6 × 10^−1^	DC
*k*_12_	6 × 10^2^	s^−1^
*k*_13_^*m*1^	1 × 10^2^	pmol L^−1^
*k*_13_^*c*1^	6 × 10^2^	pmol L^−1^ s^−1^
*k*_13_^*m*2^	1 × 10^2^	pmol L^−1^
*k*_13_^*c*2^	6 × 10^4^	pmol L^−1^ s^−1^
*k*_14_	3.6 × 10^2^	s^−1^
*k*_15_^*i*^	5	pmol L^−1^
*k*_15_	3.6 × 10^2^	s^−1^
*k*_16_	3.6 × 10^2^	s^−1^
*k*_17_^*m*1^	1 × 10^2^	pmol L^−1^
*k*_17_^*c*1^	3.6 × 10^3^	pmol L^−1^ s^−1^
*k*_17_^*m*2^	1 × 10^2^	pmol L^−1^
*k*_17_^*c*2^	3.6 × 10^5^	pmol L^−1^ s^−1^
*k*_18_^*m*^	1.5104 × 10^3^	pmol L^−1^
*k*_18_^*c*^	4.86 × 10^3^	pmol L^−1^ s^−1^
*r*	1.17 × 10^−2^	mmol L^−1^ s^−1^
*C*_1_	8.571 × 10^−1^	DC
*C*_2_	1.429 × 10^−1^	DC

## Appendix

### Equations and Parameters Underlying the Molecular Model

In the following, the ODEs are given which determine the molecular interaction model that is shortly described in Section 2.1. Furthermore, in Table 4, the mapping of the variables *x*_1_,…, *x*_10_ to the corresponding molecular species is summarized. Finally, Table 5 lists all involved reaction parameters with their original value and unit. More details on the model can be found in [41]:

(A.1) dx1dt=k1·k1i·x10∗·C1·x10+C2k1i+x5∗·(1−x5)   −x2∗·k2·x1·x2−k3·x1,dx2dt=k4−x1∗·k2·x1·x2−k6·x2,dx3dt=x1∗·x2∗x3∗·k2·x1·x2−k5·x3,dx4dt=k7·x2−k8·x4−x5∗·k9·x4·x5   +x5∗x4∗·k10·(1−x5),dx5dt=−x4∗·k9·x4·x5+k10·(1−x5),dx6dt=k11c1·x5·(1−x6)k11m1+x6∗·(1−x6)   +k11c2·(1−x5)·(1−x6)k11m2+x6∗·(1−x6)−k12i·k12·x6k12i+M,dx7dt=k13c1·(1−x6)·(1−x7)k13m1+x7∗·(1−x7)   +k13c2·x6·(1−x7)k13m2+x7∗·(1−x7)−k14·x7,dx8dt=k15i·k15·(1−x8)k15i+x7∗·x7−k16·x8,dx9dt=−k17c1·(1−x6)·x9k17m1+x9∗·x9   −k17c2·x6·x9k17m2+x9∗·x9+k18c·x8·(1−x9)k18m+x9∗·(1−x9).
