# Supplementary material for: Identification of Crucial Parameters in a Mathematical Multiscale Model of Glioblastoma Growth
Source: Comput Math Methods Med. 2014 May 8;2014:437094. doi: 10.1155/2014/437094 (PMC4034489; doi:10.1155/2014/437094)
Supplement: Supplementary file 1 — The supplementary material provides the complete results of the sensitivity coefficient calculations for all model parameters. For each parameter modification and each of the four system endpoints the corresponding sensitivity coefficients are presented in form of figures comparable to Figure 4 of the article. [file 437094.f1.pdf]

Supplementary Material: Sensitivity Coefficients

Tina A. Schuetz   Andreas Mang   Stefan Becker   Alina Toma   Thorsten M. Buzug

1 Sensitivity coefficients and partitioning of reaction parameters

In the following the four sensitivity coefficients $S^{time}, S^{total}, S^{mig}$ and S^{prolif} are shown for each reaction parameter $k_1 - k_{18}^m$. To enable a compact, though still meaningful presentation we partitioned the 31 parameters in three distinct groups. For each parameter group the sensitivity coefficients are shown separately. Parameter group 1 includes the reaction parameters k_1, k_1^i, k_2, k_3, k_4, k_5, k_6, k_7, k_8, k_9 and k_{10}. Parameter group 2 contains the reaction parameters k_{11}^{m1}, k_{11}^{c1}, k_{11}^{m2}, k_{11}^{c2}, k_{12}^i, k_{12}, k_{13}^{m1}, k_{13}^{c1}, k_{13}^{m2}, k_{13}^{c2} and k_{14}. The parameters k_{15}^i, k_{15}, k_{16}, k_{17}^{m1}, k_{17}^{c1}, k_{17}^{m2}, k_{17}^{c2}, k_{18}^m and k_{18}^c are embraced in parameter group 3. This parameter division is also shown in Fig. S1.

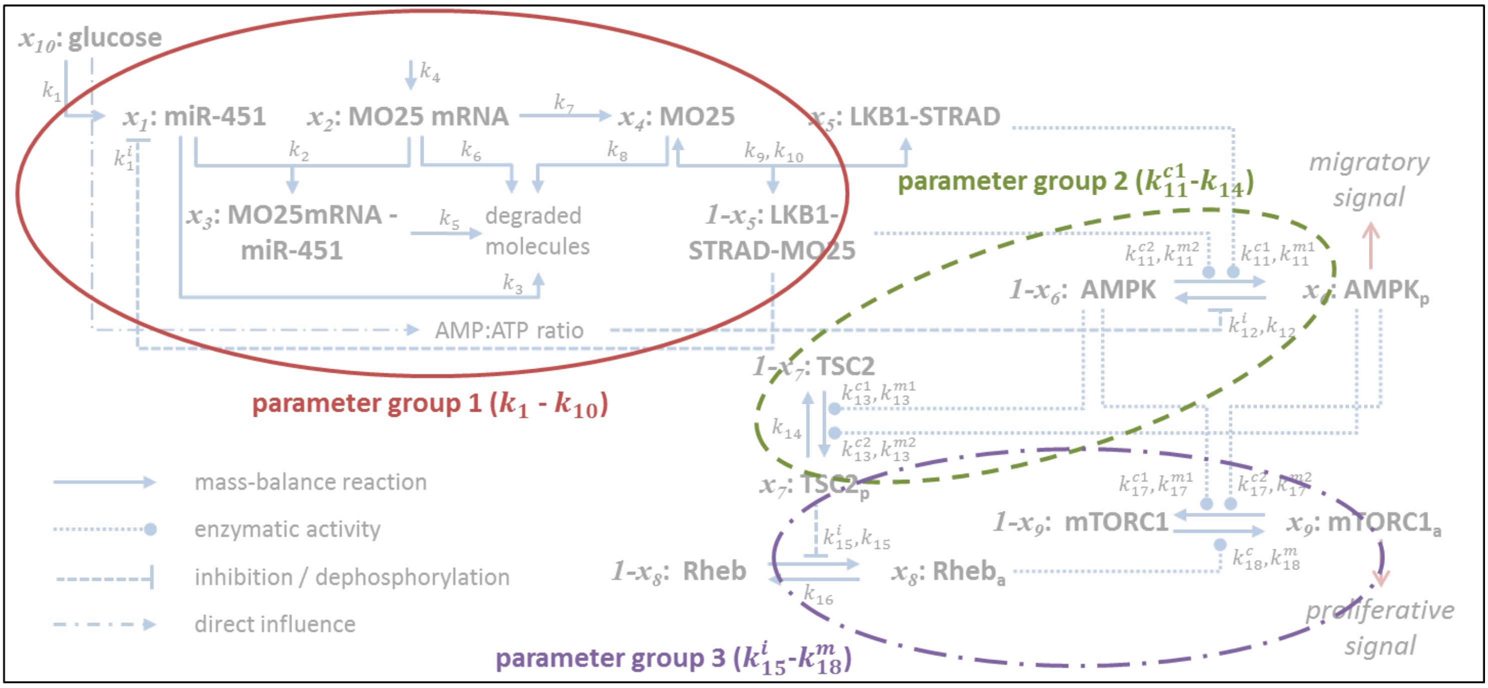

Figure S1: Partitioning of reaction parameters in three disjunct groups.

2 Parameter Overview

In Fig. S2 the sensitivity coefficients for the number of time steps S^{time} is shown for the parameters of parameter group 1. Fig. S3 displays the sensitivity coefficients for the total number of tumor cells in the last time step S^{total} for the first parameter group. Fig. S4 and Fig. S5 summarize the sensitivity coefficients for the number of migrating and proliferating cells in the last time step, S^{mig} and S^{prolif}, respectively.

The results of the sensitivity coefficients $S^{time}, S^{total}, S^{mig}$ and S^{prolif} for the second parameter group are shown in Fig. S6 - S9. The sensitivity coefficients of the parameters that are included in parameter group 3 are presented in Fig. S10 - S13.

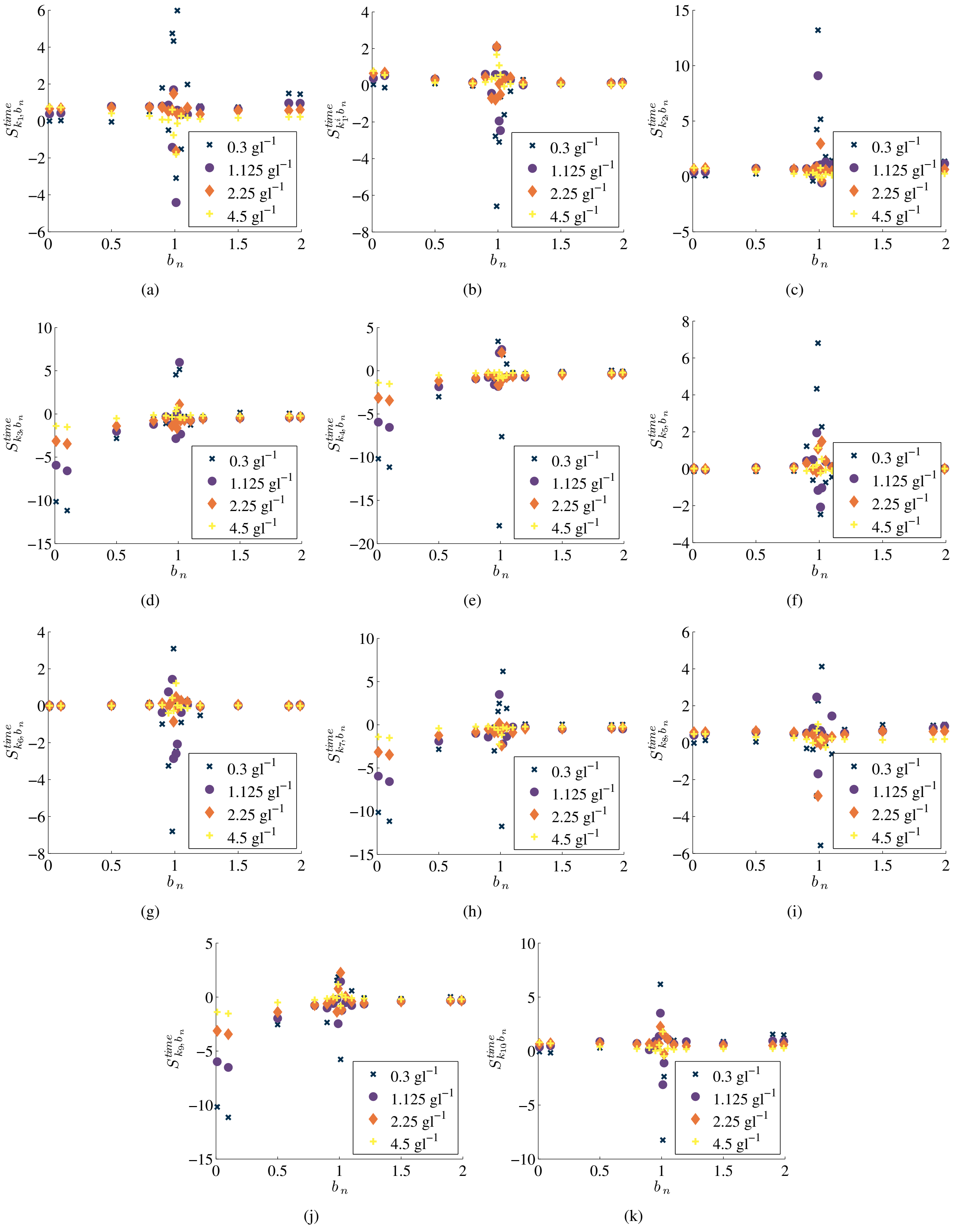

Figure S2: S^{time} for all parameters in parameter group 1.

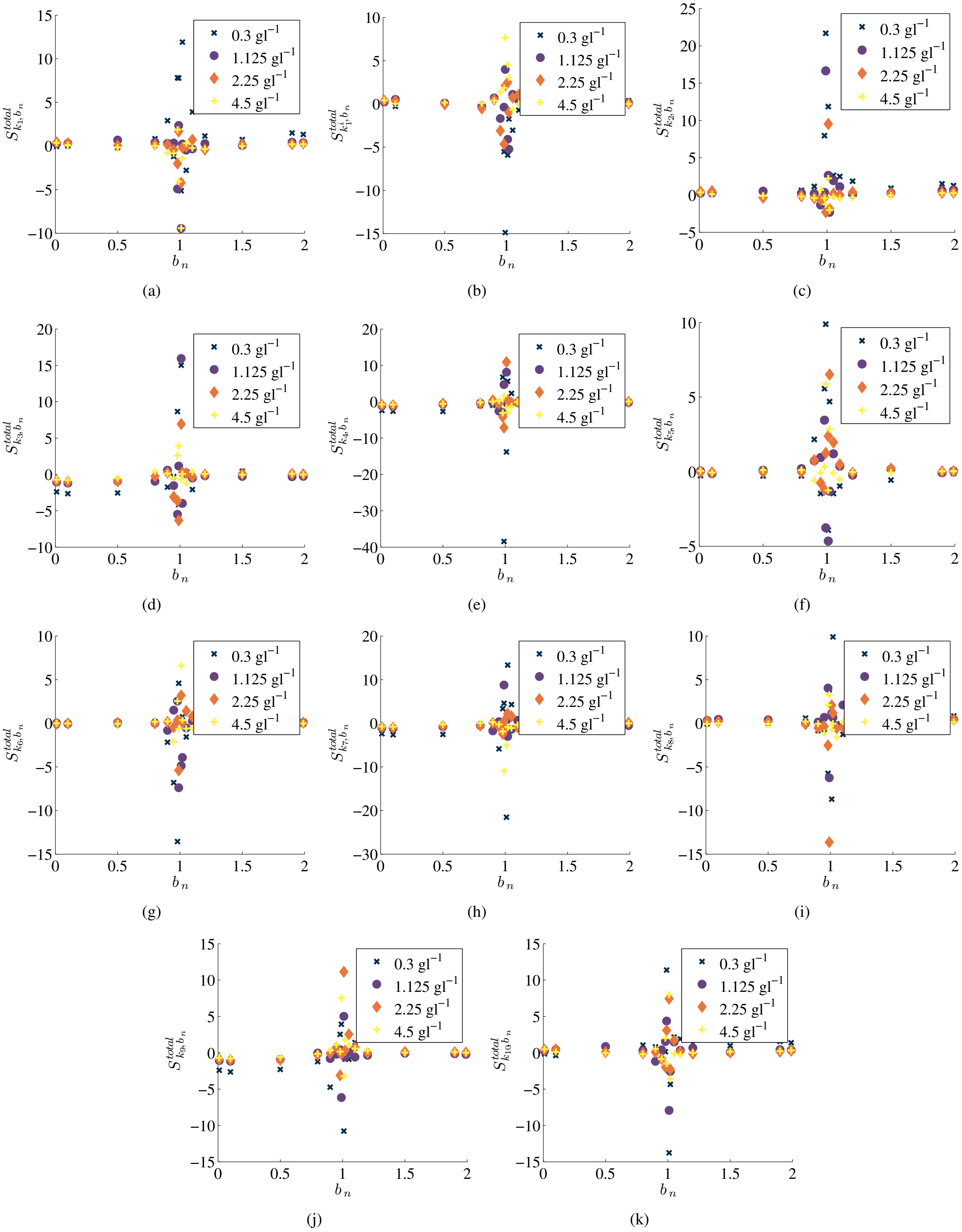

Figure S3: S^{total} for all parameters in parameter group 1.

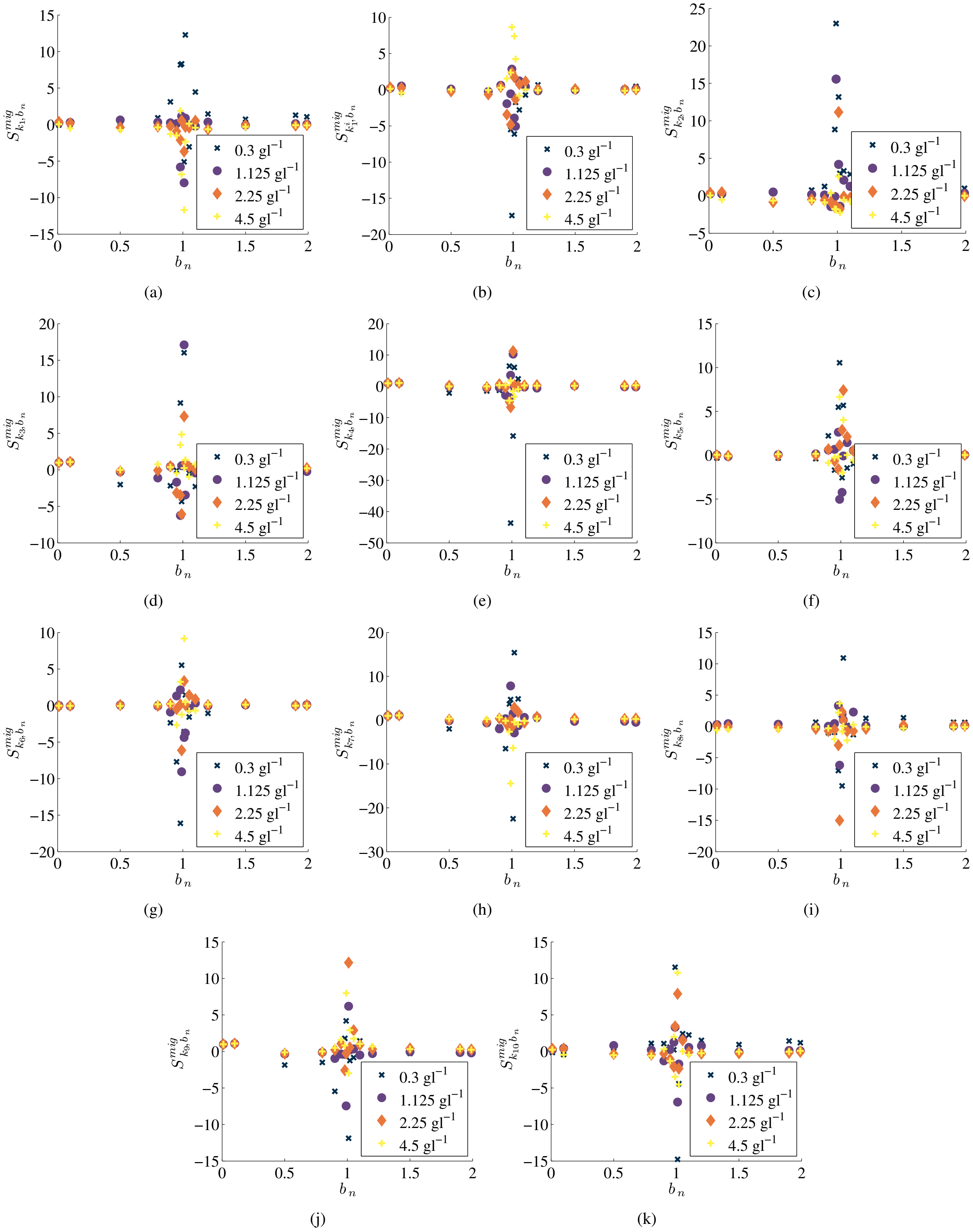

Figure S4: S^{mig} for all parameters in parameter group 1.

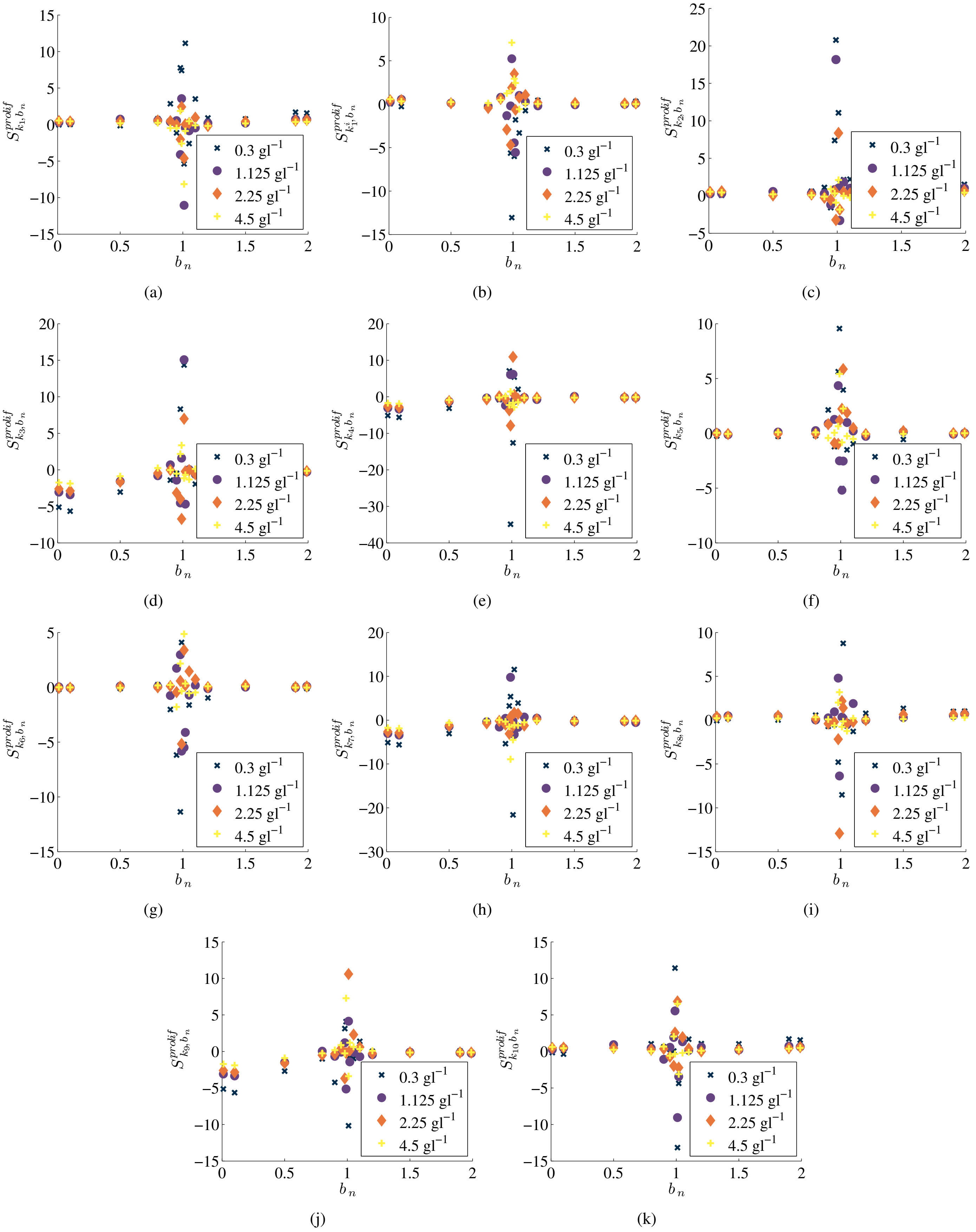

Figure S5: S^{prolif} for all parameters in parameter group 1.

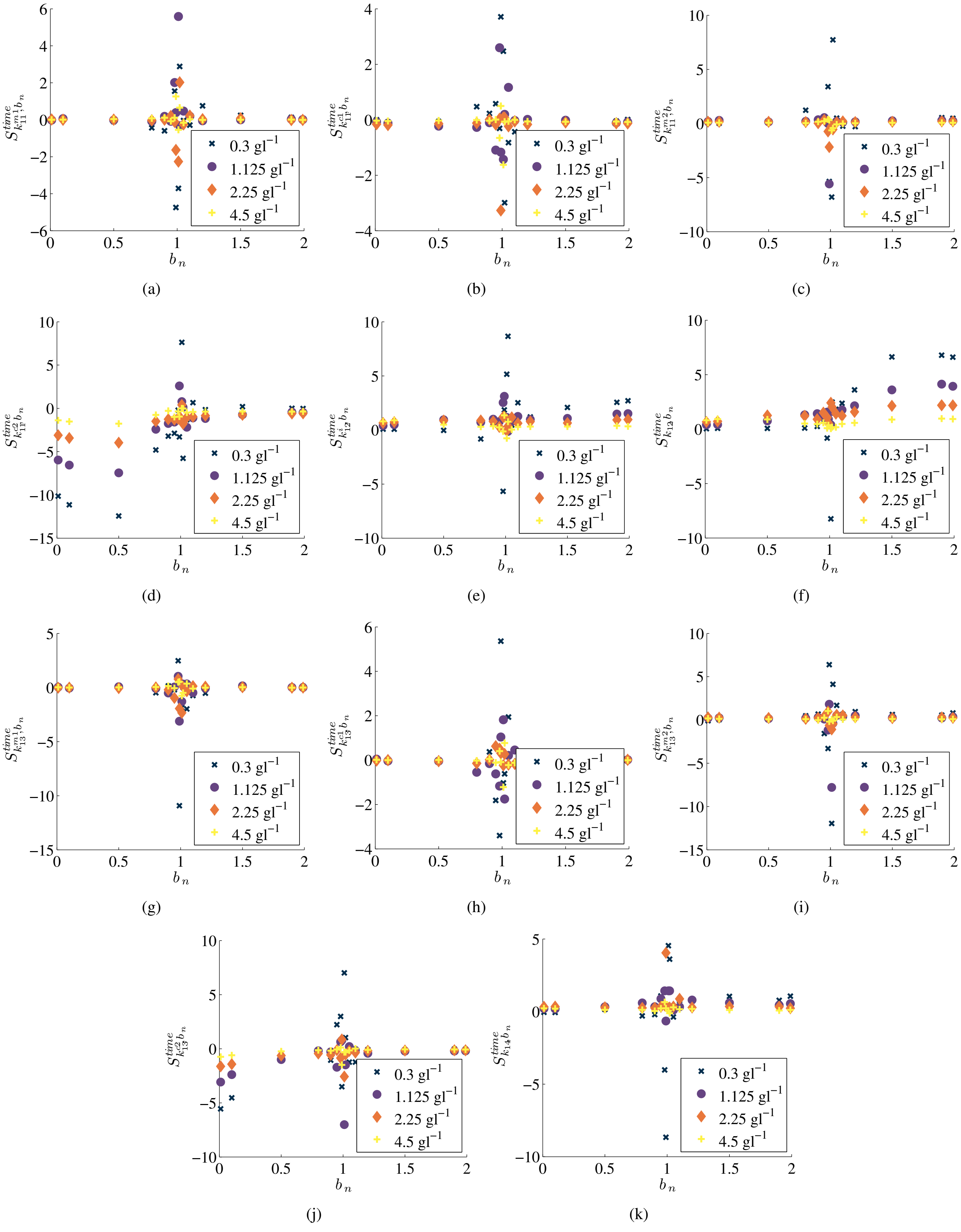

Figure S6: S^{time} for all parameters in parameter group 2.

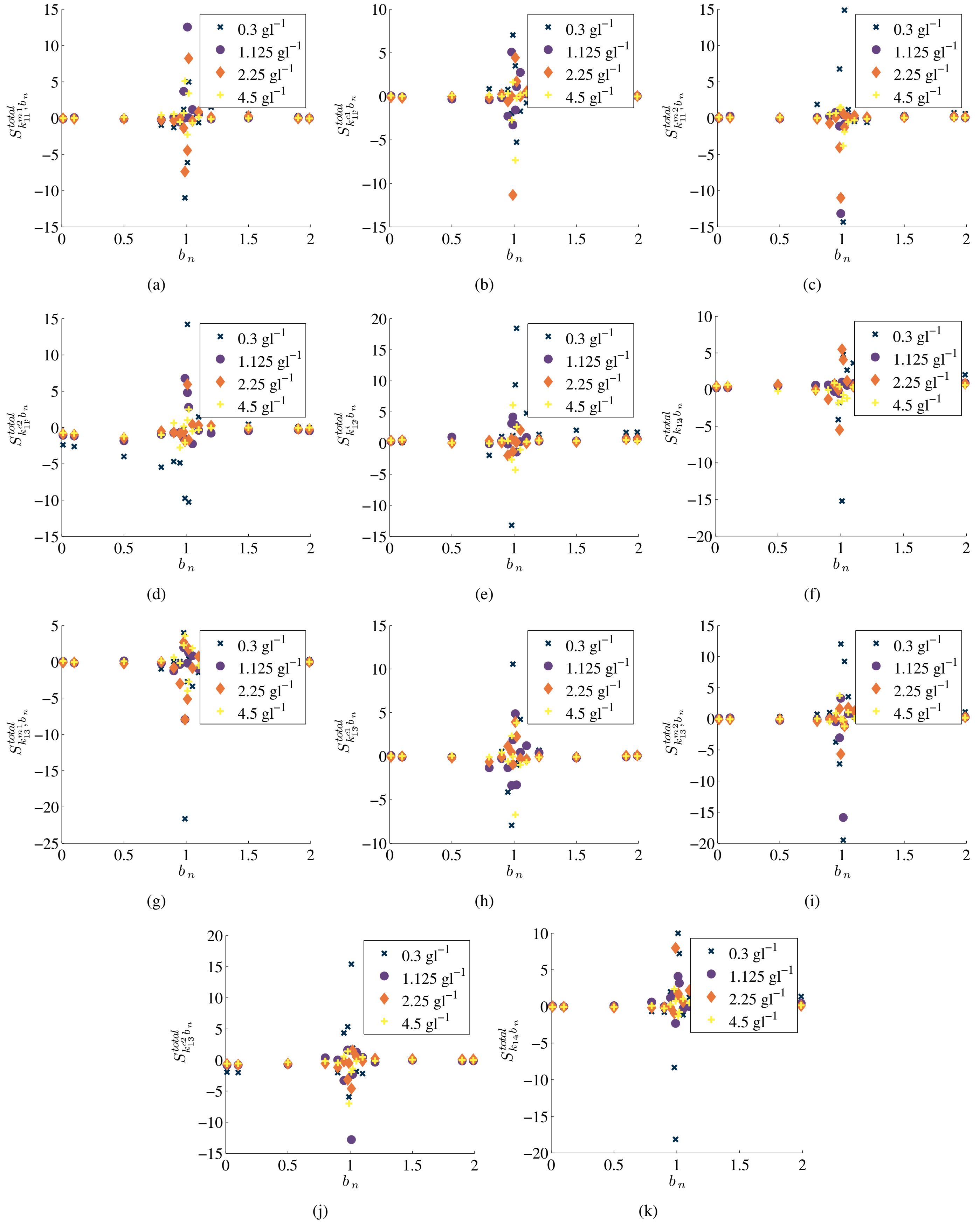

Figure S7: S^{total} for all parameters in parameter group 2.

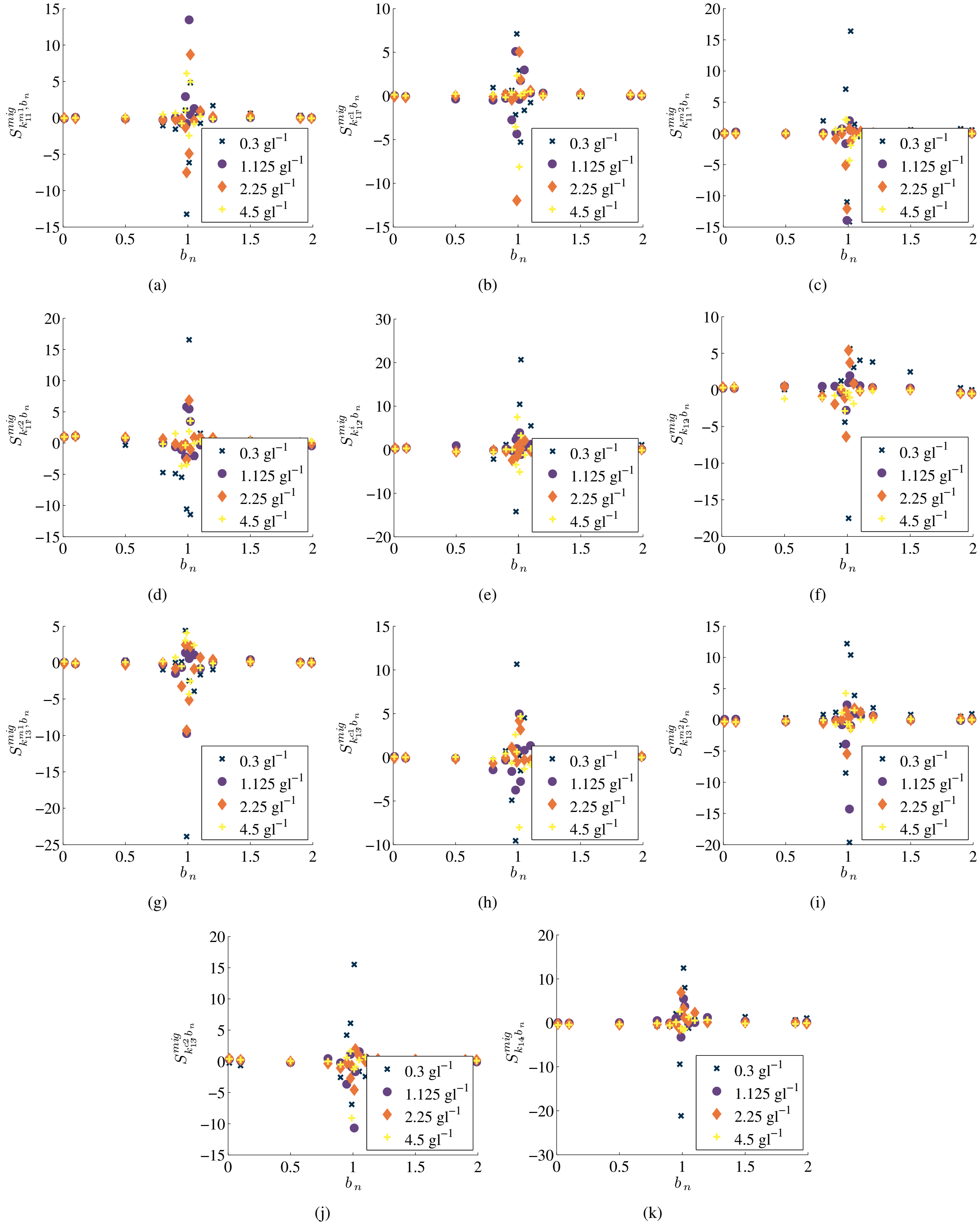

Figure S8: S^{mig} for all parameters in parameter group 2.

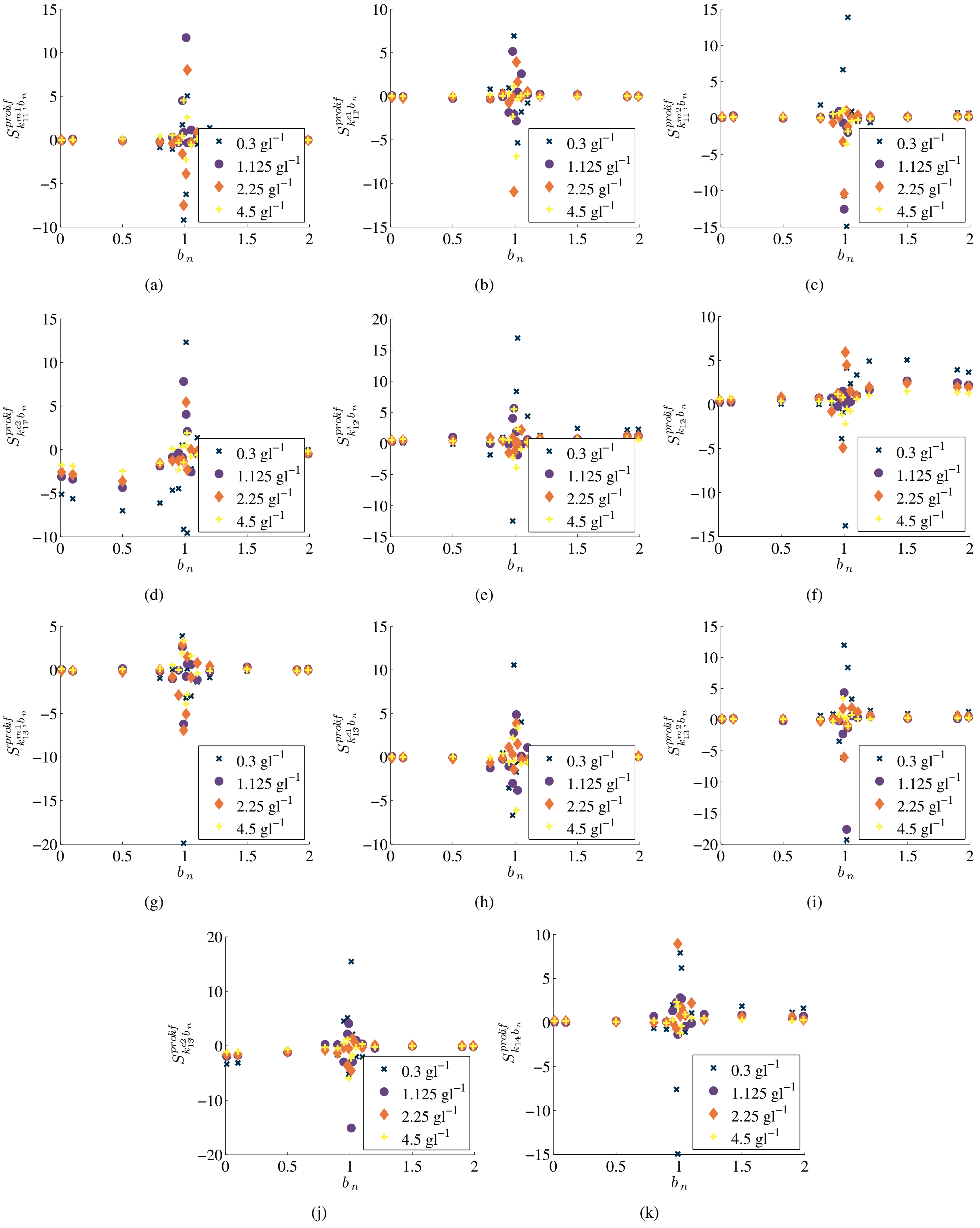

Figure S9: S^{prolif} for all parameters in parameter group 2.

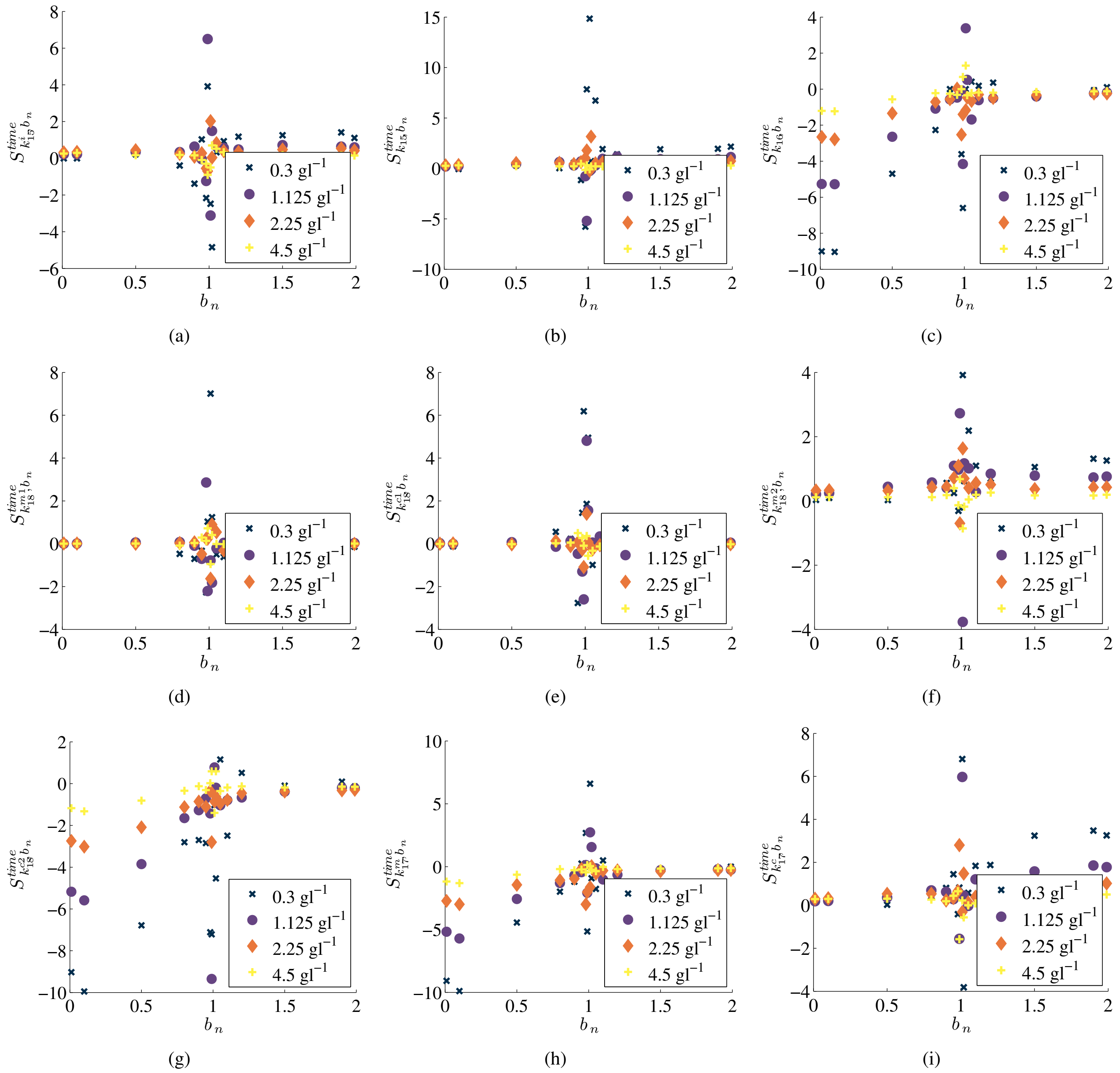

Figure S10: S^{time} for all parameters in parameter group 3.

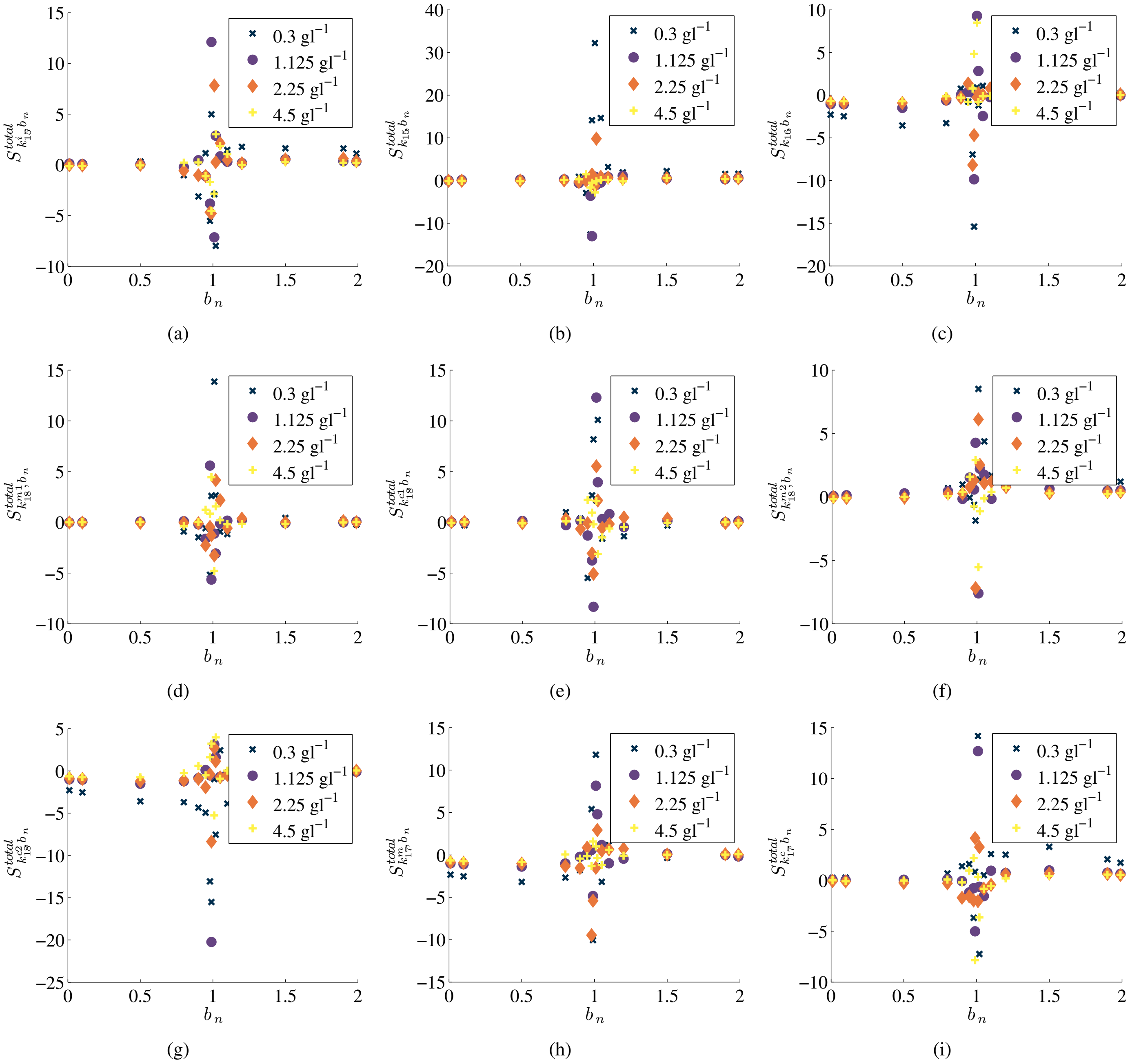

Figure S11: S^{total} for all parameters in parameter group 3.

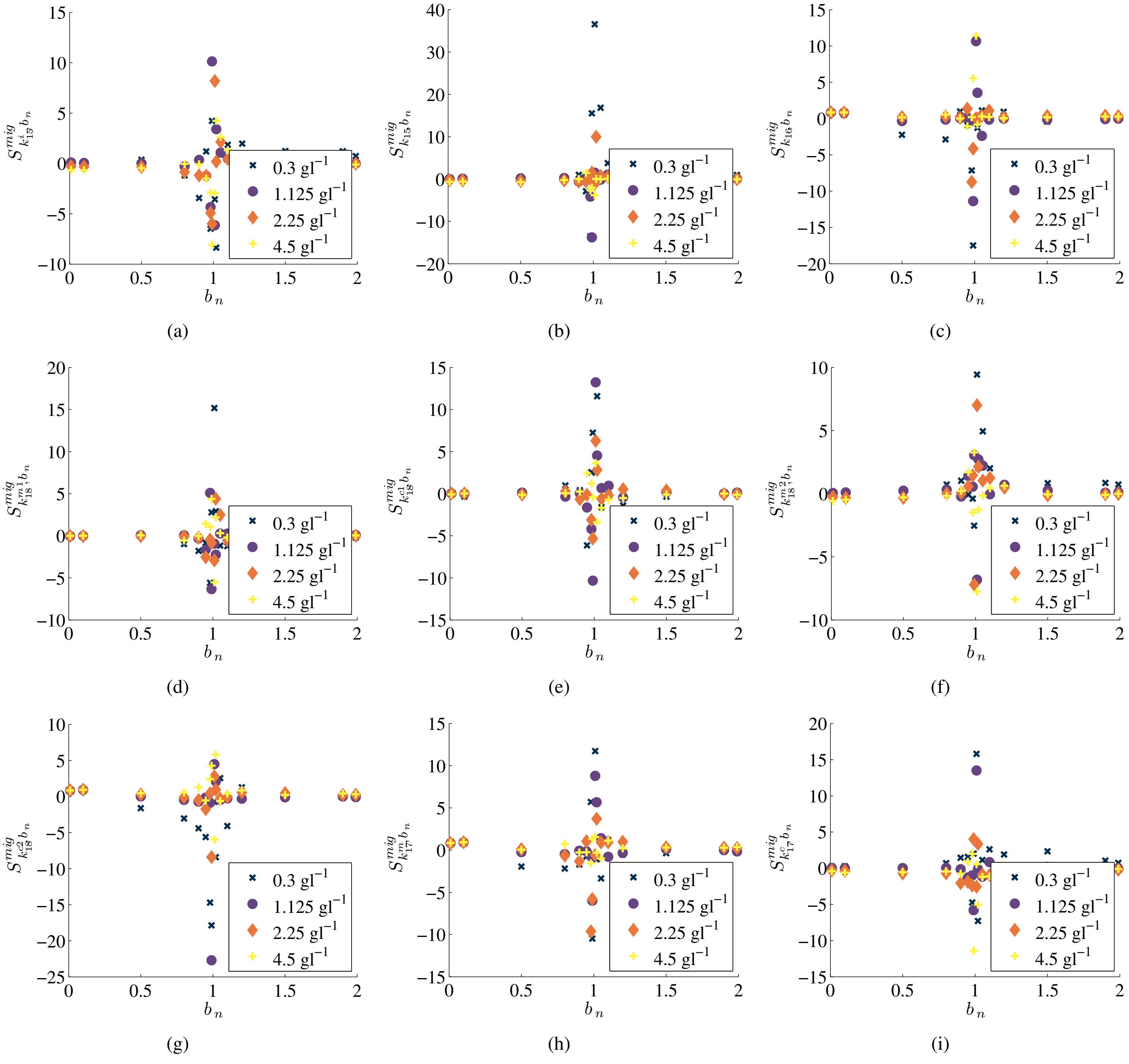

Figure S12: S^{mig} for all parameters in parameter group 3.

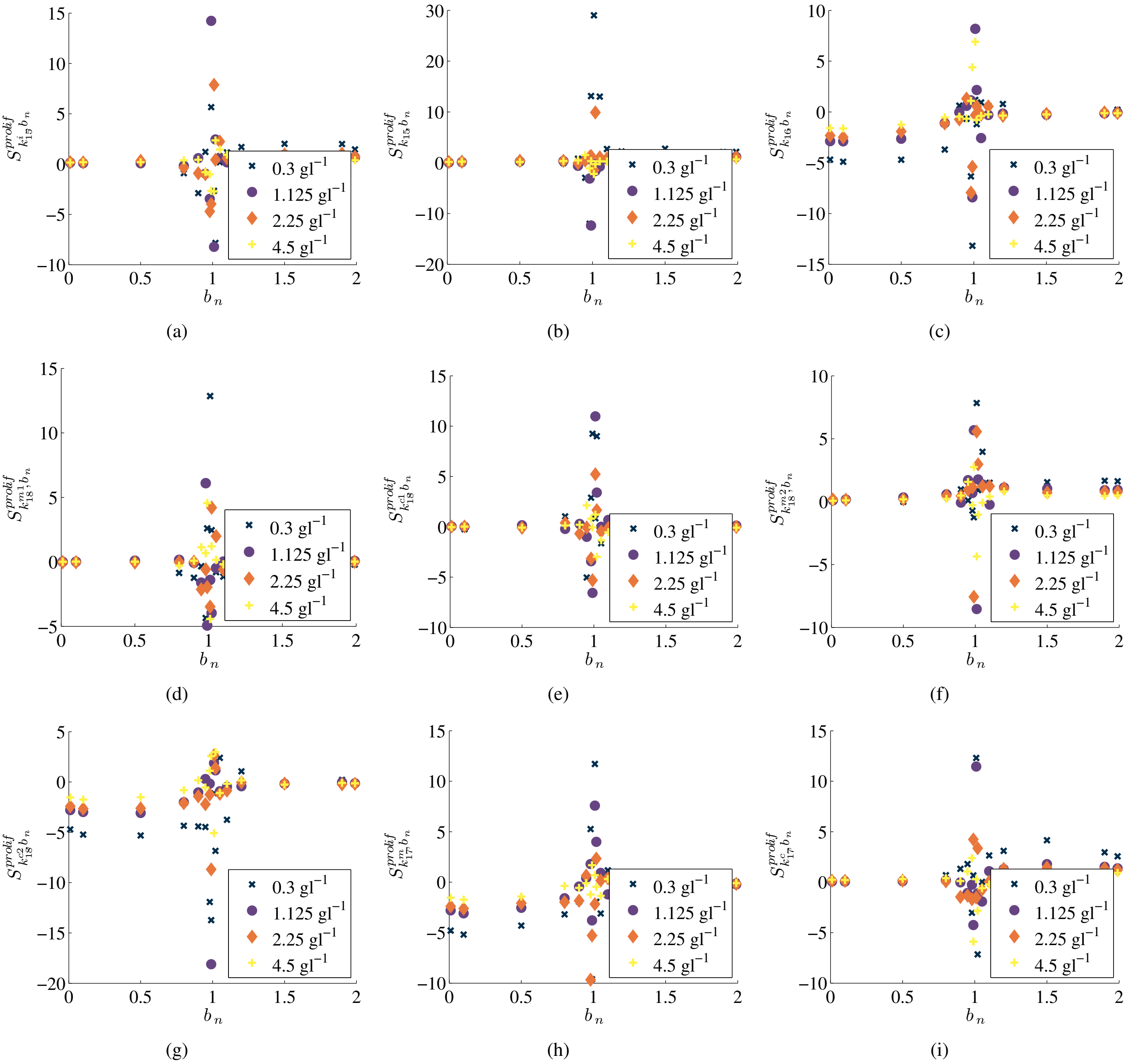

Figure S13: S^{prolif} for all parameters in parameter group 3.
